# Insights from a Long-Term Outdoor Mesocosm Study: eDNA Metabarcoding Reveals Exacerbated but Transient Impacts from a Nanoenabled Pesticide Formulation (Nano-TiO_2_-Coated Carbendazim) on Freshwater Microbial Communities

**DOI:** 10.1021/acsestwater.5c00014

**Published:** 2025-04-22

**Authors:** Martin van der Plas, Tom A. P. Nederstigt, Krijn B. Trimbos, Emilie A. Didaskalou, Martina G. Vijver

**Affiliations:** Institute of Environmental Sciences (CML), Leiden University, Leiden 2300 RA, The Netherlands

**Keywords:** community composition, nanoenabled pesticides, fungicide, field, bacteria, phytoplankton

## Abstract

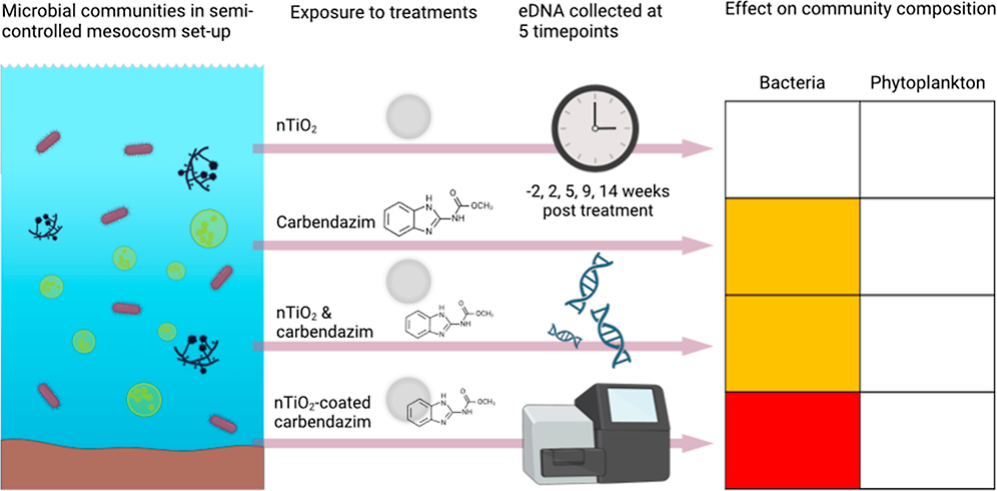

Fungicides currently encompass the second-most-used class of agricultural pesticides globally. Residues are frequently detected in freshwater, leading to undesired ecological impacts. Nanoenabled pesticide formulations have recently gained prominence in the scientific literature and have been suggested to exhibit favorable properties over conventional pesticide formulations by facilitating reductions in emissions toward nontarget locations. However, data on unintended effects on nontarget aquatic communities are scarce, especially concerning microbial communities. In this study, long-term effects of nano titanium-dioxide- (nTiO_2_)-coated carbendazim and its constituents on (pelagic) freshwater microbial communities in simulated agricultural ditches were investigated over a period of 14 weeks using environmental DNA (eDNA) metabarcoding. Impacts on bacterial diversity (α and β) were observed 2 weeks after the treatment application and most pronounced in the nTiO_2_-coated carbendazim treatment, followed by its active substance, i.e., noncoated carbendazim. The observed patterns possibly imply that nTiO_2_-coated carbendazim imposed more pronounced and potentially delayed or extended effects compared to the noncoated form of carbendazim. Bacterial communities also proved to be resilient under the tested conditions as they returned to the control-state within 5 weeks after the treatment application. Overall, our data suggest that eDNA metabarcoding data on microbial communities can help uncover time-dependent effects of nanoformulated pesticides.

## Abstract 2

Using eDNA metabarcoding, this study demonstrates exacerbated yet transient impacts of a nanoenabled pesticide formulation on freshwater microbial communities. With experimental data to this end currently being scarce, our findings can support ongoing regulatory developments.

## Introduction

Freshwater ecosystems are among the most impacted ecosystems worldwide. A significant threat to these ecosystems is posed by agricultural pesticides.^1−3^ Among these, fungicides currently encompass the second-most-used product globally, and residues are frequently detected in adjacent (nontarget) freshwater ecosystems.^4,5^ One such fungicides is carbendazim, a benzimidazole fungicide which is currently banned from use for agricultural practices in the European Union but nevertheless remains one of the most used fungicides in agriculture in some Eurasian, Latin American, and Australian regions.^6,7^ Additionally, residues of carbendazim are among the most frequently detected pesticides in surface waters both in areas where its use in agriculture is permitted and in areas where its use has been banned^5^ (see also the online tool www.bestrijdingsmiddelenatlas.nl for monitoring data on carbendazim in Dutch surface waters). In the latter case, this has been attributed to the use of other benzimidazole (pro-)fungicides (e.g., thiophanate-methyl and benomyl) of which carbendazim is a metabolite, as well as to its use as a biocidal product for nonagricultural purposes.^8^

Extensive research has documented effects of pesticides on freshwater macrofauna (e.g., see reviews^2,9,10^). However, impacts on microorganisms in these environments remain largely underexplored.^11^ Microorganisms (i.e., bacteria, fungi, phytoplankton, and protozoans) and their communities are essential to ecosystem functioning, contributing to nutrient cycling, organic matter decomposition, and overall water quality.^12^ Consequently, understanding how pesticides affect microbial communities is vital for assessing their broader ecological consequences on freshwater ecosystems.^13^

Historically, studying effects of stressors on microbial communities has predominantly relied on culturing and colony counting and the assessment of a variety of functional end points (i.e., microbially mediated processes such as respiration and decomposition rates). These methods can provide general insights into impacts on community-level end points and processes but are also known to suffer from limitations that may produce data that is biased (i.e., due to the presence of nonculturable taxa/strains) and lacks taxonomical resolution (i.e., when measuring solely functional end points).^14,15^ In recent years, the use of environmental DNA (eDNA) meta-barcoding has emerged as a favorable alternative approach to this end. eDNA metabarcoding allows for the simultaneous and noninvasive sampling and detection of a wide variety of taxa, including those that may not be detected through conventional methods. Moreover, eDNA metabarcoding allows for the rapid processing of many samples, increasing the resolution of the obtained data. Additionally, the approach requires minimal specialist knowledge for taxonomic identification, making it a cost-effective and efficient tool for large-scale environmental assessments.^16^ In the case of microbial communities, this can allow for obtaining data with a high taxonomic resolution, which in turn can help identify impacts of stressors which may not be observable through culturing methods (e.g., due to nonculturable taxa/strains) or functional assessments (e.g., due to functional redundancy).

Over the past few years, nanoenabled pesticide formulations have gained attention in the scientific literature and have been suggested to exhibit favorable properties over conventional pesticide formulations regarding efficiency and emission-reduction.^17^ Nanoenabled pesticides, consisting of nanomaterials which carry/encapsulate and deliver pesticidal active substances (ASs) in a responsive (for example, controlled, targeted, and synchronized) manner, have been suggested to offer new opportunities to increase pesticidal efficacy and efficiency, and in turn reduce environmental impacts, when compared to conventional pesticides. Various authors have argued that the functionalities that are achieved through nanoenabled formulations may also exacerbate the environmental impacts associated with its AS and overall product.^18,19^ However, to date, only a hand-full of papers have been published on potential nontarget impacts of nanoenabled pesticides on microbial communities, and these have mainly focused on terrestrial communities (for instance, see refs (20–25)).

The aim of the current study was to investigate long-term effects of a nanoformulated pesticide, nTiO_2_-coated carbendazim, and its constituents on natural freshwater nontarget microbial communities using environmental DNA (eDNA) metabarcoding. The experiment took place in a mesocosm facility consisting of test systems which simulate agricultural ditches, over a period of 14 weeks during which sampling took place 2 weeks before and 2, 5, 9, and 14 weeks after treatment application. The experiment was performed in parallel with the work reported in ref (26), which assessed the effects of the same stressors on macroinvertebrate communities through conventional sampling methods. A nanoenabled formulation of carbendazim with controlled-release properties was synthesized for the purpose of the experiment. By evaluating nontarget impacts of nano-TiO_2_-coated carbendazim as well as its separate constituents, we additionally performed a realistic and long-term assessment of impacts of nTiO_2_, which is currently among the most used and emitted nanomaterials with antimicrobial properties, on freshwater microbial communities.

## Materials and Methods

### Experimental Setup

The experiment was conducted in a series of outdoor mesocosm systems at the “Living Lab” research facility of Leiden University, The Netherlands (see http://mesocosm.org for an extensive description of the experimental site). The mesocosms’ dimensions were 5–0.8–0.3 m (length–width–depth, volume = 1200 L), which mimics dimensions commonly observed for agricultural drainage ditches. The outdoor facility is prone to fluctuations in weather conditions and was colonized prior to the experiment (over a period of three months) by natural communities consisting of macrophytes, macrofaunal, and microbial species (vertebrate species were caught and excluded from experiments). The systems were subsequently isolated from the pond by placement of acrylic barriers to prevent any exchange of organisms, treatments, and water during the experimental period, and communities were left to settle for an additional month before treatments were applied. Water quality parameters were measured over the course of the experiment to monitor the similarity between the experimental ditches. Temperature, pH, dissolved oxygen conductivity, NH_4_^+^ and NO_3_^–^, chlorophyll A, and turbidity were measured every week using a Hach HQ40d multimeter (Hach Ltd., Colorado, USA), a Vernier LabQuest 3 multimeter (Vernier Software & Technology, Oregon, USA), and a AquaFluor hand-held fluorometer (Turner Designs, Inc., San Jose, USA) and are reported in Figure S1.

Treatments consisted of a novel nTiO_2_-coated formulation of carbendazim, and its individual (i.e., nTiO_2_ and carbendazim applied separately) and combined (i.e., nTiO_2_ and carbendazim applied simultaneously) constituents, applied according to a full factorial block design, with 7 replicates per treatment and 7 control replicates (note that 5/7 replicates were sampled for eDNA). nTiO_2_-coated carbendazim was synthesized using pulsed chemical vapor deposition, by which a nanoscale layer of TiO_2_ was deposited on the surface of a dry carbendazim powder. Upon contact with water, this layer of nTiO_2_ acts as a diffusion barrier, resulting in the gradual and prolonged release of carbendazim to the surrounding environment. This type of formulation, in which ASs that are currently already in use in conventional agricultural practices, are delivered through nanoscale carrier systems, constitutes the majority of nanoenabled pesticides reported in literature to date.^17^ Treatment concentrations were selected to resemble environmentally realistic concentrations of the AS (carbendazim) as well as the carrier material (nTiO_2_). To ensure comparability, treatments in which nTiO_2_ and carbendazim were applied as individual constituents received nominal concentrations equivalent to those of the treatments consisting of the nTiO_2_-coated formulation of carbendazim. Carbendazim (CAS no. 10605-21-7, 97% purity, Sigma-Aldrich, Missouri, USA) was applied at a nominal treatment concentration of 4 μg L^–1^, aiming to achieve a time-weighted-average (TWA) concentration of 0.6 μg L^–1^ throughout the experiment. This resembles the maximum permissible annual average surface water concentrations as set for The Netherlands (BKMW, 2009) and surface water concentrations measured in various places in the world by e.g., refs (8, 27, and 28). nTiO_2_ (JRCNM01005a, European Commission—DG JRC, also provided by Degussa/Evonik as AEROXIDE P25) was applied at a nominal treatment concentration of 20 μg L^–1^, approximating the higher-end of modeled^29^ and measured (0.2–450 μg L^–1^; refs (30 and 31), respectively) concentrations of anthropogenically derived TiO_2_ in European and North-American surface waters. We previously reported a comprehensive overview of measurements of abiotic parameters and synthesis, characterization, application, and analysis of all treatments in the experimental setup in ref (26). Summarized fate data are provided in Figure S2.

### eDNA Sampling, Extraction, Amplification, and Sequencing

Environmental DNA (eDNA) samples were collected from five replicates per treatment and at five consecutive time points, i.e., 2 weeks prior to treatment application and 2, 5, 9, and 14 weeks post treatment application. Each sample consisted of 500 mL, of which 300 mL was used for filtering. Filter membranes (0.45 μm polyether sulfone) were stored in 700 μL of CTAB at −20 °C until extraction. DNA extraction and precipitation were performed as described in ref (32) and final resuspension of the pellet was performed in 100 μL of AE buffer (Qiagen, Venlo, The Netherlands).

Three different markers were used to assess community effects on three different taxonomic groups, being 16S for bacteria, 18S for phytoplankton, and ITS2 for fungi (see Table S1 for primer details). Dual-indexed Illumina amplicon libraries were prepared using a two-step PCR protocol. Initial PCRs were performed in triplicate, which were pooled after cleaning. After the second PCR, products were quantified, equimolarly pooled and cleaned. The 18S and ITS2 pools were sequenced in one run of Illumina MiSeq (flow cell PE300), the 16S pool was sequenced on a separate run (flow cell PE300), both at BaseClear BV (Leiden, The Netherlands). For a comprehensive overview of amplification and sequencing methods, see the Supporting Information.

### Bioinformatics and Data Preparation

Bioinformatics were performed with QIIME 2 2021.11.^33^ Primers and their reverse complements were trimmed from both ends, and any untrimmed reads without a primer present were discarded. For the ITS2 data, a 50 bp minimum length filter was applied as well to remove spurious, very low-length sequences. Next, paired-end fastq files were turned into merged, denoised, chimera-free, inferred sample sequences, after which reads were truncated based on interactive quality plots except for the ITS2 data where no truncation was performed due to the varying length of the ITS2 region (https://benjjneb.github.io/dada2/ITS_workflow.html). The amplicon sequence variants (ASVs) generated were assigned to taxonomy against the SILVA SSURef database (qiime release 132^34^) for bacteria and phytoplankton and against the UNITE database for fungi (qiime release 10.05.2021^35^). Sequencing reads were annotated down to the species level. Bycatch taxa (i.e., taxa other than those specifically targeted in the primer design) and unassigned sequences were removed, creating a data set with only assigned taxa of interest. Data were assessed based on both annotated ASVs (family for the 16S data, order for the 18S data, and phylum for ITS2 data) as well as unassigned ASVs. To ensure in the latter case that ASVs belonged to the desired taxonomic groups, we only included ASVs that had been assigned to at least the phylum level for the 16S and 18S data and the kingdom level (i.e., fungi) for the ITS2 data. A comprehensive report of the bioinformatics process can be found in the Supporting Information.

### Statistical Analysis

All statistical analyses were performed in R version 4.2.1.^36^ To investigate sequencing depth per sample, rarefaction curves were generated for each marker using the *vegan* package.^37^ Samples that did not reach or near a plateau were discarded, and data were rarefied based on the lowest read count to control for uneven sequencing depth. Bycatch taxa were removed and ASVs occurring only once across the data sets were removed to reduce noise of possible false positives. While singletons were not actively discarded, these were removed as a consequence of other filtering steps.

Relative read abundance (RRA) was used to visualize community composition and detect potential shifts between treatments. Although eDNA metabarcoding does not directly produce abundance data, microbial read abundance can serve as a proxy for actual microbial abundance.^38^ RRA represents the proportion of reads assigned to a given taxon relative to the total reads within a sample. However, due to the nature of metabarcoding, RRA does not provide direct information about absolute abundance changes. An observed increase in RRA for a taxon could result from its actual increase, a decrease in other taxa, or a combination of both. Additionally, differences in amplification efficiency or sequencing depth among samples may influence RRA, rendering it a relative measure rather than an absolute quantification of community changes.^39^ Therefore, RRA should primarily be used as an indicator of potential shifts rather than a definitive measure of taxon abundance.

We used both α- and β-diversity metrics to assess effects of the applied treatments on community composition. α-Diversity metrics quantify diversity within a single community or sample, whereas β-diversity provides a relative measure of diversity from one community or sample compared to another.^40^ For α-diversity, we compared taxonomic richness and Shannon–Weiner index (Shannon diversity) scores between treatments and time points. Richness is defined as the number of unique taxa, while Shannon diversity accounts for both the number and the evenness of taxa, incorporating read abundance data. To examine the effect of treatment, time, and their interaction on taxonomic richness and Shannon diversity, linear models were fitted with the “lm” function, which were subsequently analyzed with the “anova” function, both from the *stats* package.^36^ Random effects were omitted from the models as their inclusion resulted in overfitting. For each model, the distribution of residuals was assessed, and when needed, data were transformed. When this did not yield normally distributed residuals, generalized linear models (glm) were applied using the *stats* package.^36^ Individual models for each time point and each treatment were designed and analyzed for separate assessments, following the same approach. Subsequently, the “emmeans” function^41^ was used for posthoc assessment. To control for multiple comparisons, *p*-values were adjusted using the Benjamini and Hochberg method.^42^

β-Diversity was assessed based on two different indices, being the Sørensen dissimilarity index and the Bray–Curtis dissimilarity index. The first measures community (dis)similarity based on species presence/absence and thus provides a measure of (dis)similarity in taxonomic composition between compared communities. The second approach considers both species presence/absence and abundance (i.e., read abundance), thereby providing insights into the (dis)similarity in taxonomic composition and evenness between communities. Dissimilarity scores for both the Sørensen and Bray–Curtis indices were calculated using the *vegan* package.^37^ Effects of treatment and time point were assessed with permutational multivariate analysis of variance (PERMANOVA) using the “adonis2” function from the *vegan* package, with 999 permutations restricted within the blocks and replicates to account for the block design and repeated measures. After running the full factorial model, separate models per time point and per treatment were run to assess the individual effects of the treatments within each time point and time within each treatment. Again, *p*-values were adjusted following the Benjamini and Hochberg method. To test the homogeneity of dispersion, β-dispersion was calculated with the “betadisper” function in *vegan* and assessed with ANOVAs followed by Tukey’s HSD post-hoc tests.

## Results

### Exposure Characteristics and Physicochemical Water Quality Parameters

Samples collected from ditches receiving nTiO_2_ treatments initially showed water column concentrations of 23.6 ± 4.4 μg L^–1^ (mean ± standard error, background-corrected concentrations of total TiO_2_) (Figure S2A). nTiO_2_ concentrations in the water column subsequently decreased, most likely as a result of aggregation and sedimentation processes, and stabilized at 7.5 ± 0.9 μg L^–1^ between 1 week and 14 weeks after treatment application. Time-weighted average (TWA) concentrations over the entire experimental time frame in nTiO_2_ treatments were 8.8 ± 0.3 μg L^–1^.

Initial concentrations of total carbendazim (i.e., freely dissolved and nTiO_2_-coated) were 4.03 ± 0.7 μg L^–1^ in carbendazim treatments, 4.1 ± 0.3 μg L^–1^ in carbendazim and nTiO_2_ treatments and 2.9 ± 0.3 μg L^–1^ in nTiO_2_-coated carbendazim treatments. These concentrations respectively decreased to 0.3 ± 0.01, 0.5 ± 0.2, and 0.2 ± 0.1 μg L^–1^ within 1 week after treatment application (Figure S2B–D). TWA concentrations of carbendazim over the entire experimental time frame were 0.15 ± 0.01 μg L^–1^ in carbendazim treatments, 0.18 ± 0.01 μg L^–1^ in carbendazim and nTiO_2_ treatments, and 0.10 ± 0.01 μg L^–1^ in nTiO_2_-coated carbendazim treatments. For nTiO_2_-coated carbendazim treatments, discrepancies between nominal treatment concentrations and those measured immediately after treatment application may partly be attributed to aggregation and sedimentation of coated carbendazim from the water column since analysis of stock suspensions used to prepare treatments showed that the mass of carbendazim added to each ditch was equivalent to 114% of the intended treatment concentration. An extensive discussion regarding the characterization and fate of all treatments, both in the experimental setup and in vitro, is provided in ref (26).

### Descriptive Sequencing Results

A total of 4,905,205 16S reads, 1,470,463 18S reads, and 1,775,793 ITS2 reads were obtained, with an average of 39,225 ± 9010 standard deviation, 11,763 ± 3291 and 13,944 ± 4449 reads per sample, respectively. 6064 ASVs were generated for the 16S data set, 2662 ASVs for the 18S data set, and 2900 ASVs for the fungi data set. Rarefaction curves for each sample reached a plateau at ∼10,000 reads for the 16S data and at ∼6000 reads for the 18S data and ITS2 data (Figure S3). After rarefaction and removal of bycatch taxa and single occurrence ASVs, a total of 1308 bacteria ASVs, 829 phytoplankton ASVs, and 207 fungi ASVs was obtained, with an average of 169 ± 44 SD, 71 ± 24, and 12 ± 6 ASVs per sample, respectively. Bacterial ASVs were assigned to 22 phyla, 30 classes, 75 orders, and 119 families. For phytoplankton, ASVs were assigned to 13 phyla, 22 classes, and 48 orders. Fungi ASVs were assigned to 6 phyla, 10 classes, and 15 orders (Table S2). The low number of fungi taxa yielded a data set of insufficient diversity to perform robust statistical analyses on. Therefore, further assessment of the fungi data was omitted. A complete overview of sequencing results for all three markers can be found in the Supporting Information.

### Community Composition Bacteria

**Figure 1:**
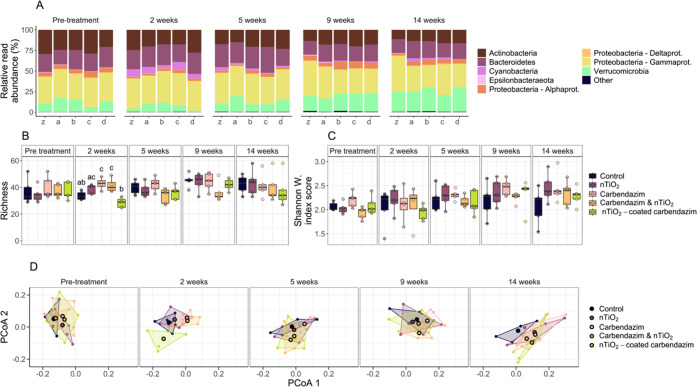
Bacterial community composition data per treatment over the course of the experiment. (A) Relative read abundance (RRA) visualized per phylum—or class within phylum for Proteobacteria—for the eight most abundant groups. Each bar represents a treatment within a time point, showing the average RRA from the five replicates. Treatments are labeled as following: z = control, a = nTiO_2_, b = carbendazim, c = nTiO_2_ and carbendazim, d = nTiO_2_-coated carbendazim; (B) boxplot visualizing family richness per treatment over time. Significant differences between treatments within time point 2 are indicated by letters, with boxes sharing a letter being not significantly different from one another; (C) boxplot visualizing read abundance-based Shannon–Weiner index scores per treatment over time. Values are based on summed reads per family; (D) PCoA plots visualizing Sørensen (dis)similarity. Centroids represent mean PCoA scores per treatment and polygons are drawn around PCoA scores of individual replicates (i.e., ditches). Boxplots and PCoAs can be found enlarged in the Supporting Information.

Over the course of the experiment, RRA across bacterial phyla was largely even between treatments within time points and to a slightly lesser extend between time points (Figure 1A). Nonetheless, some notable differences were observed among individual phyla. RRA of actinobacteria showed an overall downward trend trough time. However, at 2 weeks after the treatment application, an increase in RRA was observed in the nTiO_2_-coated carbendazim treatment compared to the previous time point as well as compared to the other treatments and the control. At the same time, a decrease in RRA of Verrucomicrobia compared to the previous time point was observed in the same treatment. This occurred in all treatments and the control in that time point, though most notable in the nTiO_2_-coated carbendazim treatment. Cyanobacteria showed an initial steep increase in RRA after 2 weeks as well for all treatments and the controls, followed by a reduction in RRA. Deltaproteobacteria, a class within the phylum Proteobacteria, showed an increase in RRA after 9 weeks, which was maintained until the end of the experiment (Figure S4).

Bacterial communities showed a statistically significant increase in richness and Shannon–Weiner index scores over the time frame of the experiment, both for unassigned (i.e., ASV-level) and assigned (i.e., family level) data (Table 1, Figures 1B,C and S5). An upward trend in both α-diversity metrics was already apparent 2 weeks after the application of the treatments for carbendazim, nTiO_2_, and combined (i.e., carbendazim and nTiO_2_) treatments. In contrast, bacterial communities in controls only increased in richness and Shannon–Weiner index scores from 5 weeks after the application of the treatments onward (Figures 1B,C and S5). Assessment of the data per treatment and time point revealed a significant effect of treatment within the two week time point on family richness (*F* = 9.77; *p* = 0.002; Table S3). Inspection of the data within the two week time point revealed significantly elevated levels of family richness in both carbendazim and combined treatments relative to controls (carbendazim: *t*_(18)_ = 3.32; *p* = 0.010; combined: *t*_(18)_ = 2.86; *p* = 0.021). In addition, bacterial richness in nTiO_2_-coated carbendazim treatments showed a notable drop from pretreatment conditions 2 weeks after the application of the treatments. Although this did not result in significant differences from controls (*t*_(18)_ = −2.05; *p* = 0.092), the effect was significant compared to all other treatments at that time point (nTiO_2_: *t*_(18)_ = −4.01; *p* = 0.003; carbendazim: *t*_(18)_ = −5.25; *p* < 0.001; combined: *t*_(18)_ = −4.79; *p* < 0.001). Based on ASV richness per phylum, most phyla followed this trend, with the exception of actinobacteria, where communities showed no change at the second time point compared to the previous time point or compared to one another (Figure S6). No significant effects were observed based on the Shannon Weiner index scores.

**Table 1:** Results from Linear Models Evaluating Taxonomic Richness and Read Abundance-Based Shannon Diversity for Bacteria and Phytoplankton over Time and across Treatments^a^

bacteria			
richness	treatment	time	interaction
ASV	*F* = 1.69; *p* = 0.157	*F* = 43.52; *p* < **0.001**	*F* = 0.29; *p* = 0.885
family	*F* = 2.91; *p* = **0.025**	*F* = 7.64; *p* = **0.007**	*F* = 0.97; *p* = 0.424
Shannon div.	treatment	time	interaction
ASV	*F* = 2.30; *p* = 0.063	*F* = 11.63; *p* < **0.001**	*F* = 0.31; *p* = 0.870
family	*F* = 2.85; p = 0.027	*F* = 21.71; *p* < **0.001**	*F* = 1.03; *p* = 0.395

Phytoplankton			
richness	treatment	time	interaction
ASV	*F* = 0.20; *p* = 0.937	*F* < 0.01; *p* = 0.968	*F* = 1.65; *p* = 0.168
order	*F* = 0.28; *p* = 0.890	*F* = 4.01; *p* = **0.048**	*F* = 0.68; *p* = 0.606
Shannon div.	treatment	time	interaction
ASV	Chisq = 0.60; *p* = 0.963	Chisq = 0.30; *p* = 0.582	Chisq = 2.42; *p* = 0.658
order	Chisq = 1.55; *p* = 0.818	Chisq = 7.69; *p* = **0.006**	Chisq = 1.82; *p* = 0.769

a For ANOVAs, *F*-values are reported; for generalized linear models, Chi-square (Chisq) values are reported. Statistically significant effects are indicated by bold *p*-values.

β-Diversity-based analyses indicated that bacterial community composition varied significantly both over time and across treatments (Figures 1D and S7, Table 2). Overall differences to this end were of similar magnitude regardless of whether analyses were performed based on assigned or unassigned data and presence and absence (i.e., Sørensen index) or read-abundance data (i.e., Bray–Curtis index, Table 2). Assessment of separate models revealed that effects of the treatments were not significant within any of the time points for either Sørensen or Bray–Curtis data, with the exception of the second time point where family-based Sørensen dissimilarity differed significantly between treatments (Table S4). Although this difference could not be statistically attributed to any specific treatment, PCoA plots showed that nTiO_2_-coated carbendazim communities differed clearly from the other communities (Figure 1D).

**Table 2:** Results from PERMANOVA Models Evaluating Differences in Sørensen and Bray–Curtis β-Diversity over Time and across Treatments for Bacterial and Phytoplankton Communities^a^

bacteria			
Sørensen	treatment	time point	interaction
ASV	*R*^2^ = 0.04; *F* = 1.52; *p* = **0.001**	*R*^2^ = 0.23; *F* = 8.66; *p* = **0.001**	*R*^2^ = 0.09; *F* = 0.81; *p* = 0.952
family	*R*^2^ = 0.05; *F* = 1.77; *p* = **0.001**	*R*^2^ = 0.22; *F* = 8.24; *p* = **0.001**	*R*^2^ = 0.1; *F* = 0.91; *p* = 0.596
Bray–Curtis	treatment	time point	interaction
ASV	*R*^2^ = 0.04; *F* = 1.30; *p* = **0.001**	*R*^2^ = 0.19; *F* = 6.90; *p* = **0.001**	*R*^2^ = 0.09; *F* = 0.80; *p* = 0.973
family	*R*^2^ = 0.04; *F* = 1.44; *p* = **0.001**	*R*^2^ = 0.16; *F* = 5.78; *p* = **0.001**	*R*^2^ = 0.10; *F* = 0.85; *p* = 0.824

Phytoplankton			
Sørensen	treatment	time point	interaction
ASV	*R*^2^ = 0.03; *F* = 1.23; *p* = **0.001**	*R*^2^ = 0.23; *F* = 8.55; *p* = **0.001**	*R*^2^ = 0.09; *F* = 0.83; *p* = 0.973
Order	*R*^2^ = 0.03; *F* = 1.42; *p* = **0.001**	*R*^2^ = 0.29; *F* = 12.13; *p* = **0.001**	*R*^2^ = 0.09; *F* = 0.94; *p* = 0.443
Bray–Curtis	treatment	time point	interaction
ASV	*R*^2^ = 0.03; *F* = 1.11; *p* = **0.001**	*R*^2^ = 0.16; *F* = 5.39; *p* = **0.001**	*R*^2^ = 0.10; *F* = 0.85; *p* = 0.984
Order	*R*^2^ = 0.02; *F* = 0.72; *p* = **0.001**	*R*^2^ = 0.23; *F* = 8.48; *p* = **0.001**	*R*^2^ = 0.08; *F* = 0.68; *p* = 0.995

a Statistically significant effects are indicated by bold *p*-values.

### Community Composition Phytoplankton

**Figure 2:**
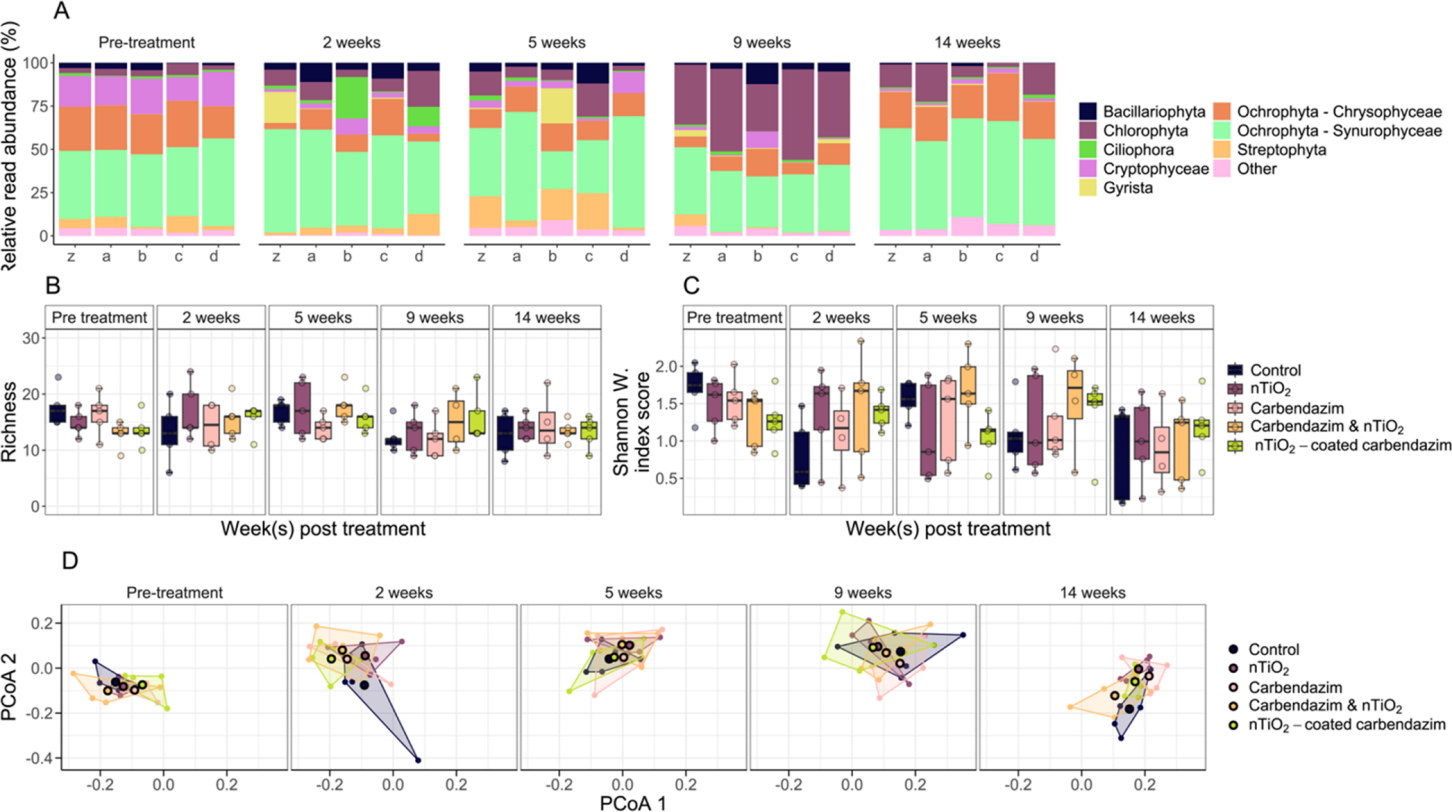
Phytoplankton community composition data per treatment over the course of the experiment. (A) Relative read abundance (RRA) visualized per phylum—or class within phylum for Ochrophyta—for the eight most abundant groups. Each bar represents a treatment within a time point, showing the average RRA from the five replicates. Treatments are labeled as following: z = control, a = nTiO_2_, b = carbendazim, c = nTiO_2_ and carbendazim, d = nTiO_2_-coated carbendazim; (B) boxplot visualizing order richness per treatment over time; (C) boxplot visualizing read abundance-based Shannon–Weiner index score per treatment over time. Values are based on summed reads per order; (D) PCoA plots visualizing Sørensen similarity. Centroids represent mean PCoA scores per treatment and polygons are drawn around PCoA scores of individual replicates (i.e., ditches). Boxplots and PCoAs can be found enlarged in the Supporting Information.

RRA varied over the course of the experiment between treatments, specifically 2 and 5 weeks after treatment application, with Ochrophyta—and specifically Synurophyceae—dominating throughout the experiment (Figure 2A). Analysis at the phylum level revealed substantial variance between samples, even within specific treatments and time points, with RRA values ranging from zero to nearly 100% within a single treatment/time point combination (Figure S8).

Prior to treatment application, phytoplankton communities exhibited notable but statistically insignificant variation in richness and Shannon–Weiner index scores (Figures 2B,C and S9). This variation could not be linked to a particular phylum, as most phyla exhibited considerable differences in richness (Figure S10). Although differences between treatments in this regard were most pronounced when α-diversity metrics were calculated on the basis of unassigned (i.e., ASV-level) data, assigned (i.e., order-level) data showed largely similar patterns. Both richness and Shannon–Weiner index scores showed a decrease relative to pretreatment conditions over the subsequent course of the experiment (Figure 2B,C, Table 1). Notably however, this could not be attributed to the application of the treatments since phytoplankton communities developed according to similar trends across treatments and controls (Table S5).

In line with observations from α-diversity metrics, analysis of β-diversity indexes indicated that phytoplankton communities varied in composition over time, when analyzed on the basis of both order and ASV data (Table 2, Figure 2D). However, in contrast with α-diversity, a statistically significant difference in β-diversity between treatments was observed before the treatment application based on binary order data (Table S6). β-Diversity-based analyses additionally showed that community composition varied across treatments, although differences in this regard were not substantial enough to be attributable to specific time points or contrasts between treatments via posthoc analyses. Analyses in which read abundance was accounted for (i.e., analyses based on Bray–Curtis dissimilarity) showed minimal differences from those solely based on the presence and absence of ASVs (or orders), indicating that differences in community composition over time and between treatments could largely be attributed to the latter (Table 2).

## Discussion

In this study, we assessed the effects of a nanoformulated pesticide, nTiO_2_-coated carbendazim and its constituents on nontarget microbial communities in a mesocosm setup consisting of replicated freshwater ditches. Impacts on community composition were observed and most pronounced in nTiO_2_-coated carbendazim treatments, followed by its AS (i.e., noncoated carbendazim). Notably, statistically significant impacts were only observed on bacterial communities and only at 2 weeks after the application of the treatments. No significant effects were observed on other microbial communities or at other time points. The absence of statistically significant differences from controls in conventional (i.e., noncoated) carbendazim treatments imply that bacterial communities experienced a more pronounced effect of the nanoformulated pesticide compared to its AS. It also shows that within the tested conditions, bacterial communities were able to recover to control levels swiftly.

### Effects on Microbial Communities

In the current study, nTiO_2_-coated carbendazim induced more pronounced effects on bacterial community composition than its constituents, manifesting specifically as a higher degree of dissimilarity (both in terms of reduced α-diversity and increased β-diversity) from control communities or other treatments. Notably, the effects on bacterial community composition observed in this study were only present 2 weeks after the application of the treatments, and differences from controls were not observed anymore at the subsequent sampling moment (i.e., 5 weeks post treatment application). No treatment-specific effects were observed on phytoplankton communities. The effects of the treatments on bacterial communities manifested in a different direction, with a decreasing trend in richness in the nTiO_2_-coated carbendazim treatment and an increasing trend in the other treatments (Figures 1B and S5). This observed difference in magnitude and direction of impacts between the treatments might be explained by the extended release of carbendazim from its nanoenabled formulation (see Supporting Information, Figure 2D), as discussed previously in ref (26). As a result of this, exposure times to carbendazim in the experimental setup may have been extended,^43^ which consequently could have delayed recovery times of bacterial communities. It is conceivable that in the time up to the first sampling moment, communities in other carbendazim-containing treatments were similarly affected but subsequently recovered as a result of more rapid dissipation of carbendazim from the system. The observed increase in taxonomic richness compared to the control may in that case be explained by the fact that communities in early stages of succession are often characterized by a higher species richness compared to those in subsequent stages.^44,45^ Moreover, the observed change in bacterial community composition in the combined treatment was similar to that of the noncoated carbendazim and not to nTiO_2_-coated carbendazim. This suggests that the observed effects of the nTiO_2_-coated formulation of carbendazim were distinct from those of its constituents and did not result solely from their combined application. To gain a more comprehensive understanding of these temporal dynamics, additional sampling points, particularly those closer to the treatment date, would be beneficial. Such data could help clarify the early stage responses of microbial communities and provide further insights into the effects of the dissipation patterns of nTiO_2_-coated carbendazim and its constituents. Nonetheless, the findings from the current study highlight the potential for nanoenabled formulations to have unique, time-dependent impacts on bacterial communities, and emphasize the importance of evaluating nanoenabled pesticides independently from their individual components to sufficiently understand their effects on nontarget organisms and communities. Moreover, the transient nature of the impacts of both nTiO_2_-coated carbendazim and conventional carbendazim observed in the current study suggests that bacterial communities can recover from exposure to both, which is in concurrence with previous observations from studies on the effects of conventional carbendazim on microbial communities in terrestrial systems.^46−48^

While antimicrobial properties of both carbendazim and nTiO_2_ have been amply described in literature (e.g., see refs (48–52)), their potential implications for freshwater bacteria and their functions in aquatic ecosystems have received relatively little attention to date. For carbendazim, observed antimicrobial properties of relevance to freshwater ecosystems have mainly relied on measures of microbially mediated processes. For example, the authors of ref (51) demonstrated that carbendazim can reduce rates of microbially mediated leaflitter decomposition in freshwater mesocosms, and comparable observations have been reported in ref (50). Notably, however, both studies only observed impacts at test concentrations that were respectively >250 and 50 times higher than those applied in the current study. Several studies have similarly shown that nTiO_2_ can negatively affect microbially mediated decomposition rates as well as the development of microbial biomass, although again at treatment concentrations that generally far (i.e., >1000 times) exceed those in the current experiment.^49,53−55^ Similarly, previous studies found that both compounds can adversely affect phytoplankton, although observations in this regard have again been reported only at higher concentrations compared to the ones applied in this study (e.g., see refs (56 and 57) for effects of nTiO_2_ and refs (58−60) for effects of carbendazim on phytoplankton).

In the current study, we only observed effects of the treatments when analyzing bacterial community data using occurrence-based metrics, (i.e., taxonomic richness and Sørensen (dis)similarity), not abundance-based metrics (i.e., Shannon Weiner index score, Bray–Curtis (dis)similarity, or RRA). Within bacterial communities, abundant strains usually account for a large portion of total (read) abundance while only comprising a small fraction of the total number of strains.^45^ In contrast, rare strains tend to make up the majority of the strains present in a community while only accounting for a small portion of the total (read) abundance.^45,61,62^ As in the current study, no effects were observed based on read abundance but only on taxa occurrence, it is likely that mainly rare strains were affected by the treatments. Opposed to abundant strains, rare strains often have specialist functions and contribute less to common functional processes—such as carbon cycling.^63^ As such, it can be expected that no change in function could be observed at the low concentrations applied in this study, explaining why we did observe effects based on community composition data, and studies that focused on functional end points did not (i.e., at lower concentrations). In contrast with observations for bacterial communities, the current study showed no effects of any of the individual treatments on phytoplankton communities. It should be noted that variance in the phytoplankton community data may have partly masked effects in this regard since full models of both Sørensen- and Bray–Curtis-based β-diversity did indicate an overall treatment effect on phytoplankton community composition, which could not be attributed to specific treatments or time points due to insufficient statistical power in posthoc analyses, most likely compounded by within-treatment variability in community composition. Variability in the phytoplankton community composition between mesocosms assigned to different treatments was also observed prior to their application (primarily when analyzed as order-based Sørensen dissimilarity), and between-replicate (or within-treatment) variation may be considered an inherent trade-off when aiming to establish realistic mesocosm studies.^64^ In the current case, it is, however, unlikely that aside from reducing statistical power, this may have significantly affected the response of phytoplankton communities to the applied treatments since data from subsequent sampling moments provided no clear indication of differing temporal trends in community development between treatments.

### Ecological Implications of Observed Effects

The current study showed that effects on microbial communities varied in severity and direction between nTiO_2_-coated- and noncoated carbendazim, and we have previously reported effects of both treatments on macroinvertebrate communities observed in the same experimental setup (assessed through conventional sampling, i.e., morphological identification).^26^ However, as discussed in ref (26), macroinvertebrate communities were found to be unable recover within the time frame of the experiment, while the findings from the current study show recovery of all affected communities within 5 weeks after the application of the treatments. Possibly, this difference can be attributed to a different generational turnover pace between macroinvertebrates and micro-organisms. While reproduction cycles of micro-organisms generally comprise only several hours or days, most macroinvertebrates take anywhere from several weeks to years to reproduce.^65^ This shorter generation time can allow microbial communities to respond more rapidly to changing conditions compared to macroinvertebrates, e.g., through adaptive gene expression or evolutionary adaptation, and to restore rapidly once conditions return to the control state since reproduction can start directly when conditions are favorable. Moreover, microbial taxa are able to disperse more easily over short distances, allowing them to recolonize an ecosystem.^66^ This is especially likely in a setup like the one used in the current study, where test systems are in close proximity of one another. As such, effects of stressors such as carbendazim might be noticeable only as long as microbial communities are being exposed, while for macroinvertebrates, effects could remain observable for a prolonged period.

As microbial taxa play key roles in ecosystem functioning such as decomposition, it may be expected that cascading or indirect effects can occur as a result of changes in community composition, either through top-down or bottom-up processes.^10,67^ In ref (26), we reported an overall decrease in macroinvertebrate biomass (i.e., abundance multiplied by average taxa-specific mass) in all carbendazim treatments relative to controls as well as to the earlier sampling time points, indicating a (faster) decrease in living organisms in the carbendazim treatments compared to the controls. Subsequently, this could have resulted in an increase in the substrate for microbiota to decompose, potentially allowing decomposing taxa to thrive. In contrast, the decrease in (shredder) macroinvertebrate biomass could have resulted in a decrease in the available substrate surface area for microbiota to decompose. However, in the current study, we observed no change in RRA simultaneous to the reported decrease in macroinvertebrate taxa reported in ref (26), making it unlikely for such a cascading effect to have contributed to the observed effects. Moreover, effects on bacteria coincided with treatments (i.e., decreasing concentrations of carbendazim over time) and not with macroinvertebrate community restoration (which was not observed within the time frame of the experiment), further arguing against a top-down cascading effect. Lastly, as effects on microbial taxa differed between treatments and those on macroinvertebrates did not (at least for the carbendazim containing treatments), a bottom-up cascading effect is also unlikely to have occurred. This suggests that the observed effects were predominantly driven by direct sensitivity of bacteria and macroinvertebrates to the applied treatments and highlights that for transient stressors such as carbendazim, microorganisms and macroinvertebrates could potentially serve best as bioindicators for shorter- and longer-term impacts, respectively.

The findings of the current study indicate that the nanoformulated pesticide nTiO_2_-coated carbendazim exhibits different effects compared to its individual constituents. To understand the mechanistic drivers of this difference (i.e., whether it is caused by a delayed or extended exposure, or whether other factors may play a role), further research with a more refined sampling scheme may be beneficial. Our results underscore the necessity of distinguishing between nanoenabled pesticides and conventional formulations when assessing their environmental implications. This is especially relevant in realistic environmental settings, where complex interactions may differ from controlled laboratory conditions. Advances in environmental DNA and metabarcoding techniques provide powerful tools for detecting and monitoring microbial responses, enabling more comprehensive studies on the ecological impacts of both pesticides and nanomaterials.

## Conclusions

In the present study, we used environmental DNA metabarcoding to investigate effects of a nanoenabled pesticide formulation (i.e., nTiO_2_-coated carbendazim) and its constituents on microbial communities. Low exposure concentrations of nTiO_2_-coated carbendazim, which were representative of those of conventional (i.e., noncoated) carbendazim observed in natural freshwaters, significantly impacted bacterial communities under realistic environmental conditions. Bacterial communities were affected to a lesser extent by the AS (i.e., noncoated carbendazim) than by the nTiO_2_-coated formulation. Phytoplankton communities were not significantly affected by any of the treatments throughout the experiment. The observed patterns indicate that for bacterial communities, nTiO_2_-coated carbendazim imposed a more pronounced effect, possibly caused by a delayed or extended effect, compared to conventional (i.e., noncoated) carbendazim, which nevertheless proved transient over the experimental time frame.

## Supporting Information Available

Additional details on methods, sequencing results, primer details, taxonomic annotation results, statistical output, physiochemical water quality parameters and treatment concentrations over the experimental duration, rarefaction curves, RRA plots, and α- and β-diversity plots (PDF)
