# Supplementary material for: Insights from a Long-Term Outdoor Mesocosm Study: eDNA Metabarcoding Reveals Exacerbated but Transient Impacts from a Nanoenabled Pesticide Formulation (Nano-TiO2-Coated Carbendazim) on Freshwater Microbial Communities
Source: ACS ES T Water. 2025 Apr 22;5(5):2421–31. doi: 10.1021/acsestwater.5c00014 (PMC12070410; doi:10.1021/acsestwater.5c00014)
Supplement: Supplementary file 1 — ew5c00014_si_001.pdf [file ew5c00014_si_001.pdf]

Supporting information

Insights from a long-term outdoor mesocosm study: eDNA metabarcoding reveals exacerbated but transient impacts from a nano-enabled pesticide formulation (nano-TiO_2-coated carbendazim) on freshwater microbial communities

Martin van der Plas*[a], Tom A.P. Nederstigt[a], Krijn B. Trimbos[a], Emilie A. Didaskalou[a], Martina G. Vijver[a]

[a] Institute of Environmental Sciences (CML), Leiden University, Leiden 2300 RA, The Netherlands

* Corresponding author:

E-mail address: m.van.der.plas@cml.leidenuniv.nl

<u>Total pages: 22</u>

<u>Number of tables: 6</u>

<u>Number of figures: 11</u>

Table of contents

Supporting information - Methods ... 3

Sampling and extraction of environmental DNA ... 3

DNA amplification and MiSeq sequencing ... 3

Bioinformatics and data preparation ... 4

Supporting information - Results ... 6

Sequencing results 16S ... 6

Sequencing results 18S ... 6

Sequencing results ITS2 ... 7

Supporting tables ... 8

Supporting figures ... 13

References ... 26

Supporting information – Methods

Sampling and extraction of environmental DNA

Environmental DNA (eDNA) samples were collected from five replicates per treatment and at five consecutive time points, i.e., 2 weeks prior to treatment application, and 2, 5, 9, and 14 weeks post treatment application. Each sample consisted of 500 mL of water collected from 10 subsamples obtained evenly across the length and depth of each mesocosm system. Samples were stored in sterilized Nalgene bottles and filtered on-site within 2 hours of collection. Filtration of 300 mL of each sample was performed in sterilized Nalgene filter units (Thermo Fisher, Waltham, Massachusetts, United States) connected to a vacuum pump with 0.45 µm polyethersulfone (PES) filter membranes (Merck KGaA, Darmstadt, Germany). While for microbial assessment a smaller pore size might be preferred due to cell size, we opted for the 0.45 µm filters due to the turbid nature of the mesocosms, as a finer pore size would result in a decrease in filtered water volume. Consequently, it is possible that a fraction of the microbial communities is not captured. However, studies have suggested that in turbid water, filtering a larger volume can be as important as, or more important than, using a finer pore size for capturing a substantial part of the community [1,2]. An earlier study in the same mesocosm facility that used 0.45 µm filters successfully assessed microbial communities and were able to distinguish communities exposed to treatments [3]. Moreover, within this study we did not aim to assess the total diversity. Instead, we aimed to capture a representable fraction of the community through the mesocosm that we could compare between treatments. Filter membranes were stored in 700 µl CTAB at -20°C until extraction. DNA extraction and precipitation was performed as described in ref. [4] and final resuspension of the pellet was performed in 100 µl AE buffer (Qiagen, Venlo, the Netherlands).

DNA amplification and MiSeq sequencing

Three different markers were used to assess community effects on three different taxonomic groups: a 273 bp fragment of the 16S rRNA for bacteria [5], a ± 400 bp fragment of 18S rRNA V4 subregion for diatoms and other phytoplankton [6] and a 330 bp fragment of ITS2 for fungi [7]. For all markers, the primers included 5' Illumina tails (see SI Table S1 for complete primer sequences). The primers for the 18S rRNA V4 region were originally designed for diatom studies, but proved to cover a broad range of phytoplankton taxa in an earlier study in the same experimental facility [4]. For all 125 samples (25 replicate ditches, five time points) and each marker, PCR reactions were performed in triplicate. To assess cross-contamination and tag jumping between samples during the amplification, the PCR plates for the ITS2 marker included two samples of DNA extracted from fungal specimens unlikely to occur in the setup (*Imleria badia* and *Amanita frostiana*).

Dual-indexed Illumina amplicon libraries were prepared using a two-step PCR protocol. Initial PCRs were performed in 25 µl reactions containing 1X KAPA Taq ReadyMix with dye (Roche, Basel, Switzerland), 0.5 µM of each primer and 2.0 µl of 1:10 diluted template DNA. Initial denaturation was performed at 95°C for 3 min, followed by 35 cycles at 95°C for 30 s, 50°C for 30 s and 72°C for 1 min, followed by final elongation at 72°C for 1 min. PCR products were checked on 2% precast agarose E-gels™ with SYBR™ Safe (Invitrogen, Thermo Fisher Scientific Inc.). Triplicates were combined and then cleaned with a one-sided size selection using NucleoMag NGS-Beads (Macherey-Nagel, Düren, Germany), in a 1:0.9 ratio (sample/beads). Dual-index PCRs were performed using 2.0 µl of PCR product from the first round in a 20 µl reaction containing 1x KAPA HiFi HotStart ReadyMix (Roche) and 1.5 µl of each primer (Nextera XT Index Kit v2, Illumina, San Diego, California, United States). Initial denaturation was performed at 95°C for 3 min, followed by 8 cycles at 95°C for 30 s, 55°C for 30 s and 72°C for 30 s, followed by final elongation at 72°C for 5 min. PCR products were quantified on a TapeStation system 4200 (Agilent Technologies, Santa Clara, California, United States) with a D1000 ScreenTape Assay and samples were pooled equimolarly for each marker separately. The pools were cleaned with a one-sided size selection using NucleoMag NGS-Beads in a 1:0.9 ratio and quantified again. The fungi and phytoplankton pools were combined equimolarly and sequenced in one run of Illumina MiSeq (flow cell PE300), the bacteria pool was sequenced on a separate run (flow cell PE300), both at BaseClear BV (Leiden, the Netherlands).

Bioinformatics and data preparation

Bioinformatics were performed with QIIME 2 2021.11 [8]. Primers and their reverse complements were trimmed from both ends using *qiime cutadapt trim-paired* (minimum match 5 bp, maximum error rate 0.2) [9] and any untrimmed reads without a primer present were discarded. For the ITS2 data a 50bp minimum length filter was applied as well to remove spurious, very low-length sequences. The *qiime dada2 denoise-paired* pipeline (default settings) was used to turn the paired-end fastq files into merged, denoised, chimera-free, inferred sample sequences [10]. Reads were truncated based on interactive quality plots when quality would drop (bottom of the box below q20 and mean below q27) at the end of the read, except for the ITS2 data were no truncation was performed due to the varying length of the ITS2 region (https://benjjneb.github.io/dada2/ ITS_workflow.html). To allow for more reads to pass the filtering step on the ITS2 data, since no truncation was applied, the maximum number of expected errors (maxEE) on the reverse reads was relaxed to 4 (instead of the default 2). The amplicon sequence variants (ASVs) generated were assigned to taxonomy using the *q2-feature-classifier classify-sklearn* naïve Bayes taxonomy classifier [11,12] against the SILVA SSURef database (qiime release 132 [13]) for bacteria and phytoplankton, and against the UNITE database for fungi (qiime release 10.05.2021 [14]). The default 0.7 confidence threshold for limiting taxonomic depth

was used on all data except for the ITS2 sequences as they seem to perform best with a 0.9 confidence threshold [11]. To train the classifiers, simulated amplicon reads based on our primer sites were extracted from the 16S (for bacteria) and 18S (for phytoplankton) at 99% identity representative sequences of the SILVA database with *qiime feature-classifier extract-reads*. A minimum of 200 bp and a maximum of 500 bp length was also applied to exclude simulated amplicons that were far outside of the anticipated length distribution using these primers and classifiers were further trained with *qiime feature-classifier fit-classifier-naive-bayes* [12]. As QIIME 2 developers advise, fungal ITS2 classifiers trained on the UNITE reference database do not benefit from extracting reads to primer sites, so our classifier was trained on the full reference developer sequences of the UNITE database.

For each marker, the dataset was adjusted based on control samples to account for contamination and tag/sample jumping. Based on the positive controls in the ITS2 dataset, a threshold of 0.03% was calculated which was subsequently applied to remove any ASV that accounted for less than that percentage of the total reads within a sample to account for tag jumping. We assume this is a consistent process between all PCR plates and therefore we applied this threshold on the bacteria and phytoplankton datasets as well. Next, we filtered reads based on negative controls to account for contamination during sample extraction and library-prep. This was done based on the negative control data for each marker separately. To account for contamination, we used a variation on a commonly used method [15]; any ASV with > 0.5x the highest read count in a control sample among all samples was removed.

Sequencing reads were annotated down to species level. Bycatch taxa (i.e., taxa other than those specifically targeted in the primer design) and unassigned sequences were removed, creating a data set with only assigned taxa of interest. Inspection of the annotated data revealed limited accuracy in the annotations below family level for the 16S dataset, below order level for the 18S and below phylum level for the ITS2 dataset. While ideally the lowest level of taxonomy (i.e., species for assigned taxa, or unassigned ASVs) is used for the most detailed assessment [16,17], this can also limit further analyses in practice. Analyzing data based on unassigned ASVs can introduce a high level of variance between samples due to the occurrence of many rare ASVs, which moreover is strongly influenced by bioinformatic filtering steps [18,19]. On the other hand, analyzing data based on species (or another low taxonomic) level can lead to a dataset with reduced ASVs due to incomplete reference databases [20], as was the case in the current study. To minimize the potential downsides of both methods, we assessed the data based on both annotated data (family for the 16S data, order for the 18S data and phylum for ITS2 data) as well as unassigned ASVs. To ensure that ASVs belonged to the desired taxonomic groups,

we only included ASVs that had been assigned to at least the phylum level for the 16S and 18S data and kingdom level (i.e., fungi) for the ITS2 data.

Supporting information – Results

Sequencing results 16S

A total of 8,203,274 reads were generated, with 4,268 reads originating from the 12 negative control samples. On average, each environmental sample yielded 65,592 ± 15,210 standard deviation reads. Following bioinformatic filtering in QIIME, 4,905,205 reads were retained, which generated 6,064 ASVs. Control sample correction led to the removal of 3,561 ASVs, representing 66,966 reads (1.4%). Rarefaction curves for each sample plateaued at approximately 10,000 reads (SI Fig. S3A). Two samples were excluded due to having fewer than 10,000 reads. Using the next lowest read count (19,867 reads) as the cutoff, the dataset was rarefied, removing two ASVs and 2,394,598 reads (49.5%). Taxa not assigned to the kingdoms Bacteria or Archaea were removed, followed by the removal of ASVs marked as chloroplast. This reduced the dataset by 269,929 reads (11%) and 353 ASVs, leaving 2,148 ASVs. After excluding ASVs with fewer than 2 occurrences, 1,308 ASVs remained, with an average of 169 ± 44 ASVs per sample. The ASVs were assigned to 22 phyla, 30 classes, 75 orders, and 119 families (SI Table S2).

Sequencing results 18S

For phytoplankton, the sequencing run returned a total of 3,026,616 reads, of which 1,333 reads belonged to the 12 negative controls. Per environmental sample, an average of 24,202 ± 6,488 reads were obtained. After bioinformatic filtering in QIIME, 1,470,463 reads were retained, generating 2,662 ASVs. Correction based on control samples removed 232 ASVs from the dataset, accounting for 1,827 reads (0.1%). Rarefaction curves for each sample reached a plateau at ~6,000 reads (SI Fig. S3B). Three samples were removed due to an insufficient number of reads (<6,000 reads). Based on the next lowest read sample, 6,361 reads was used as cut-off point to rarefy the dataset, which removed 16 ASVs and 692,594 reads (47.2%) from the dataset. Bycatch taxa were removed by removing any ASV assigned to the supergroup *Opisthokonta, Rhizaria*, or *Amoebozoa*. Subsequently, ASVs assigned to the phylum *Oomycota, Apicomplexa, Hyphochytriomycota, MAST, Perkinsozoa*, or to no phyla were removed as well. This resulted in a reduction of 95,069 reads (12.3%) and 1,014 ASVs, leaving a dataset with 1,400 ASVs. After removal of ASVs with < 2 occurrences in the dataset, a total of 829 ASVs were obtained, with an average of 71 ± 24 ASVs per sample. ASVs were annotated to 13 phyla, 22 classes and 48 orders (SI Table S2).

Sequencing results ITS2

Sequencing generated a total of 3,638,086 reads, with 4,700 reads originating from the ten negative control samples and 51,263 reads from the two positive controls. The average number of reads per environmental sample was 28,657 ± 8,720. After bioinformatic processing in QIIME, 1,775,793 reads were retained, generating 2,900 ASVs. Correction using control samples led to the removal of 531 ASVs, accounting for 37,940 reads (2.2%). Rarefaction analysis indicated a plateau at approximately 6,000 reads for each sample (SI Fig. S3C). Five samples were discarded due to having fewer than 6,000 reads. Using the next lowest read count (6,938) as the threshold, the dataset was rarefied, removing 56 ASVs and 890,055 reads (51.7%). Bycatch taxa were eliminated by excluding any ASV not assigned to the fungi kingdom. This resulted in the loss of 780,871 reads (93.8%) and 1,836 ASVs. The final dataset consisted of 477 ASVs. After filtering out ASVs with fewer than 2 occurrences, 207 ASVs remained, with an average of 12 ± 6 ASVs per sample. These ASVs were assigned to 6 phyla, 10 classes and 15 orders (SI Table S2).

The low yield of fungal ASVs may suggest that the ITS2 primers were not fit for the taxa present in the experimental setup. It is possible that a preferential bias of PCR reactions towards amplifying phytoplankton DNA, which comprised a large amount of the obtained ASVs in the ultimate dataset, contributed in this regard as well. After removal of these and other bycatch taxa from the dataset, the overall read abundance in the ITS2 data was considered too low and variable (either samples with very low reads had to be retained, or samples had to be discarded) to perform robust statistical analyses. Vice versa, the primers used for metabarcoding of phytoplankton also yielded fungal ASVs. Merging these ASVs with those obtained using the ITS2 primers however did not resolve the variability between replicates or low read abundance to such an extent that the overall quality of the data was deemed sufficient, and therefore further analyses of impacts on fungal community structure was omitted from the current study.

List of tables

S1. Primers for first and second PCR. 9

S2. Number of unique taxa and number of assigned ASVs obtained per taxonomic level for each marker after removal of bycatch taxa and other filtering steps. 9

S3. F-values and p-values from post-hoc assessment of ANOVA models evaluating richness and read abundance-based Shannon Weiner index scores (Shannon div.) per timepoint and treatment for bacteria. 9

S4. Results from post-hoc assessments of treatment and time effects on Sørensen and Bray-Curtis based beta-diversity from PERMANOVA analyses conducted per timepoint and treatment for bacteria. 10

S5. F-values and p-values from post-hoc assessment of ANOVA models evaluating richness and read abundance-based Shannon Weiner index scores (Shannon div.) per timepoint and treatment for phytoplankton. 11

S6. Results from post-hoc assessments of treatment and time effects on Sørensen and Bray-Curtis based beta-diversity from PERMANOVA analyses conducted per timepoint and treatment for phytoplankton. 12

Supporting table S1. Primers for first and second PCR.

PCR1			
16S	F: *TCGTCGGCAGCGTCAGATGTGTATAAGAGACAG* - CAGCMGCCGCGGTAA		Reference 1
	R: *GTCTCGTGGGCTCGGAGATGTGTATAAGAGACAG* - TACNVGGGTATCTAATCC		Reference 1
18S	F: *TCGTCGGCAGCGTCAGATGTGTATAAGAGACAG* - ATTCCAGCTCCAATAGCG		Reference 2
	R: *GTCTCGTGGGCTCGGAGATGTGTATAAGAGACAG* - GACTACGATGGTATCTAATC		Reference 2
ITS2	F: *TCGTCGGCAGCGTCAGATGTGTATAAGAGACAG* - GCATCGATGAAGAACGCAGC		Reference 3
	R: *GTCTCGTGGGCTCGGAGATGTGTATAAGAGACAG* - TCCTCCGCTTATTGATATGC		Reference 3
PCR2			
Nextera	AATGATACGGCGACCACCGAGATCTACAC - [i5 index] - *TCGTCGGCAGCGTC*		
	CAAGCAGAAGACGGCATACGAGAT - [i7 index] - *GTCTCGTGGGCTCGG*		

Note: The sequence in italic is the universal tail, the other part is the marker-specific primer.

Supporting table S2. Number of unique taxa and number of assigned ASVs obtained per taxonomic level for each marker after removal of bycatch taxa and other filtering steps.

	**Bacteria**		**Phytoplankton**		**Fungi**	
	# unique taxa	# assigned ASVs	# unique taxa	# assigned ASVs	# unique taxa	# assigned ASVs
ASV	1308	NA	829	NA	207	NA
Family	119	1044	-	-	-	-
Order	75	1200	48	586	15	57
Class	30	1260	22	765	10	58
Phylum	22	1282	13	829	6	142

Supporting table S3. F-values and p-values from individual ANOVA models assessing richness and read abundance-based Shannon-Wiener index (Shannon div.) separately for each timepoint and treatment in bacterial communities.

			Overall treatment effect per timepoint		
**Richness**	*Pre-treatment*	*2 weeks*	*5 weeks*	*9 weeks*	*14 weeks*
ASV	F = 1.05; p = 0.581	F = 2.06; p = 0.214	F = 0.16; p = 0.954	F = 0.73; p = 0.727	F = 0.36; p = 0.93
Family	F = 1.25; p = 0.869	F = 9.77; p = **0.002**	F = 2.01; p = 0.331	F = 1.69; p = 0.385	F = 0.38; p = 0.869
			Overall time effect per treatment		
	Control	*nTiO2*	*Carbendazim*	*Combined*	*nTiO2-coated CBZ*
ASV	F = 7.97; p = **0.032**	F = 16.46; p = **0.005**	F = 5.74; p = **0.04**	F = 6.3; p = **0.04**	F = 9.79; p = **0.024**
Family	F = 5.22; p = 0.106	F = 6.21; p = 0.102	F = 0.22; p = 0.869	F = 0.05; p = 0.869	F = 1.45; p = 0.401
			Overall treatment effect per timepoint		
**Shannon div**	*Pre-treatment*	*2 weeks*	*5 weeks*	*9 weeks*	*14 weeks*
ASV	F = 1.3; p = 0.515	F = 0.71; p = 0.651	F = 1.15; p = 0.515	F = 0.62; p = 0.651	F = 0.68; p = 0.651
Family	F = 2.46; p = 0.158	F = 0.42; p = 0.791	F = 0.6; p = 0.791	F = 0.77; p = 0.791	F = 1.39; p = 0.453
			Overall time effect per treatment		
	Control	*$nTiO_2$*	*Carbendazim*	*Combined*	*$nTiO_2$-coated CBZ*
ASV	F = 1.31; p = 0.515	F = 5.76; p = 0.249	F = 0.95; p = 0.515	F = 4.1; p = 0.275	F = 1.44; p = 0.515
Family	F = 0.09; p = 0.791	F = 9.01; p = **0.032**	F = 5.75; p = 0.082	F = 9.88; p = **0.032**	F = 5.15; p = 0.082

Note: Statistically significant effects are indicated by bold p-values. Combined = Carbendazim & $nTiO_2$; CBZ = carbendazim.

Supporting table S4. Results from individual PERMANOVA models assessing treatment and time effects on Sørensen- and Bray-Curtis-based beta diversity in bacterial communities, analyzed separately for each timepoint and treatment.

	Overall treatment effect per timepoint				
**Sørensen**	*Pre-treatment*	*2 weeks*	*5 weeks*	*9 weeks*	*14 weeks*
ASV	R2 = 0.157; F = 0.93; p = 0.18	R2 = 0.203; F = 1.15; p = 0.18	R2 = 0.169; F = 1.02; p = 0.314	R2 = 0.146; F = 0.86; p = 0.713	R2 = 0.146; F = 0.86; p = 0.639
Family	R2 = 0.163; F = 0.97; p = 0.224	R2 = 0.288; F = 1.82; p = **0.002**	R2 = 0.155; F = 0.91; p = 0.544	R2 = 0.202; F = 1.26; p = 0.156	R2 = 0.119; F = 0.67; p = 0.859
	Overall time effect per treatment				
	Control	*$nTiO_2$*	*Carbendazim*	*Combined*	*$nTiO_2$-coated CBZ*
ASV	R2 = 0.125; F = 3.28; p = **0.002**	R2 = 0.135; F = 3.6; p = **0.002**	R2 = 0.142; F = 3.63; p = **0.002**	R2 = 0.146; F = 3.76; p = **0.002**	R2 = 0.154; F = 4.19; p = **0.002**
Family	R2 = 0.142; F = 3.79; p = **0.002**	R2 = 0.13; F = 3.45; p = **0.002**	R2 = 0.156; F = 4.08; p = **0.002**	R2 = 0.147; F = 3.79; p = **0.003**	R2 = 0.146; F = 3.93; p = **0.002**
	Overall treatment effect per timepoint				
**Bray-Curtis**	*Pre-treatment*	*2 weeks*	*5 weeks*	*9 weeks*	*14 weeks*
ASV	R2 = 0.17; F = 1.03; p = 0.200	R2 = 0.19; F = 1.06; p = 0.330	R2 = 0.17; F = 0.99; p = 0.336	R2 = 0.14; F = 0.79; p = 0.859	R2 = 0.13; F = 0.72; p = 0.924
Family	R2 = 0.16; F = 0.97; p = 0.277	R2 = 0.16; F = 0.87; p = 0.578	R2 = 0.15; F = 0.85; p = 0.578	R2 = 0.15; F = 0.91; p = 0.578	R2 = 0.19; F = 1.17; p = 0.23
	Overall time effect per treatment				
	Control	*$nTiO_2$*	*Carbendazim*	*Combined*	$nTiO_2$-*coated CBZ*
ASV	R2 = 0.11; F = 2.73; p = **0.007**	R2 = 0.09; F = 2.29; p = **0.010**	R2 = 0.09; F = 2.11; p = **0.008**	R2 = 0.15; F = 3.76; p = **0.007**	R2 = 0.13; F = 3.53; p = **0.007**
Family	R2 = 0.13; F = 3.37; p = **0.005**	R2 = 0.1; F = 2.52; p = **0.036**	R2 = 0.09; F = 2.2; p = **0.036**	R2 = 0.14; F = 3.59; p = **0.005**	R2 = 0.13; F = 3.47; p = **0.013**

Note: Statistically significant effects are indicated by bold p-values. Combined = Carbendazim & $nTiO_2$; CBZ = carbendazim. No significant differences were observed in betadispersion.

Supporting table S5. F-values and p-values from individual ANOVA models assessing richness and read abundance-based Shannon-Wiener index (Shannon div.) separately for each timepoint and treatment in phytoplankton communities.

			Overall treatment effect per timepoint		
**Richness**	*Pre-treatment*	*2 weeks*	*5 weeks*	*9 weeks*	*14 weeks*
ASV	F = 2.5; p = 0.378	F = 0.34; p = 0.945	F = 0.34; p = 0.945	F = 0.42; p = 0.945	F = 0.15; p = 0.963
Order	F = 2.24; p = 0.502	F = 0.52; p = 1	F = 1.13; p = 0.745	F = 0.93; p = 0.775	F = 0.18; p = 1
			Overall time effect per treatment		
	Control	*$nTiO_2$*	*Carbendazim*	*Combined*	*$nTiO_2$-coated CBZ*
ASV	F = 0.49; p = 0.833	F = 0.47; p = 0.833	F = 1.5; p = 0.635	F = 3.48; p = 0.378	F = 1.37; p = 0.635
Order	F = 4.03; p = 0.502	F = 0.95; p = 0.745	F = 1.27; p = 0.745	F = 0; p = 1	F = 0.06; p = 1
			Overall treatment effect per timepoint		
**Shannon div**	*Pre-treatment*	*2 weeks*	*5 weeks*	*9 weeks*	*14 weeks*
ASV	F = 2.04; p = 0.727	F = 0.23; p = 0.966	F = 0.33; p = 0.966	F = 0.14; p = 0.966	F = 0.18; p = 0.966
Order	F = 1.31; p = 0.571	F = 1.2; p = 0.571	F = 1.54; p = 0.571	F = 0.37; p = 0.917	F = 0.21; p = 0.927
			Overall time effect per treatment		
	Control	*$nTiO_2$*	*Carbendazim*	*Combined*	*$nTiO_2$-coated CBZ*
ASV	F = 0.02; p = 0.966	F = 0.33; p = 0.966	F = 0.27; p = 0.966	F = 1.61; p = 0.727	F = 1.79; p = 0.727
Order	F = 4.34; p = 0.485	F = 2.03; p = 0.56	F = 2.36; p = 0.56	F = 0.71; p = 0.583	F = 0.12; p = 0.909

Note: Combined = Carbendazim & $nTiO_2$; CBZ = carbendazim.

Supporting table S6. Results from individual PERMANOVA models assessing treatment and time effects on Sørensen- and Bray-Curtis-based beta diversity in phytoplankton communities, analyzed separately for each timepoint and treatment.

			Overall treatment effect per timepoint		
**Sorensen**	*Pre-treatment*	*2 weeks*	*5 weeks*	*9 weeks*	*14 weeks*
ASV	R2 = 0.16; F = 0.97; p = 0.149	R2 = 0.15; F = 0.85; p = 0.88	R2 = 0.19; F = 1.14; p = 0.053	R2 = 0.15; F = 0.82; p = 0.921	R2 = 0.14; F = 0.75; p = 0.921
Order	R2 = 0.23; F = 1.53; p = **0.022**	R2 = 0.18; F = 1.05; p = 0.443	R2 = 0.17; F = 1.05; p = 0.443	R2 = 0.12; F = 0.64; p = 0.84	R2 = 0.18; F = 1.08; p = 0.443
			Overall time effect per treatment		
	Control	*$nTiO_2$*	*Carbendazim*	*Combined*	*$nTiO_2$-coated CBZ*
ASV	R2 = 0.12; F = 3.11; p = **0.002**	R2 = 0.13; F = 3.36; p = **0.002**	R2 = 0.12; F = 2.9; p = **0.002**	R2 = 0.13; F = 3.36; p = **0.002**	R2 = 0.12; F = 3.23; p = **0.002**
Order	R2 = 0.18; F = 4.91; p = **0.003**	R2 = 0.18; F = 4.92; p = **0.003**	R2 = 0.26; F = 7.3; p = **0.003**	R2 = 0.17; F = 4.42; p = **0.003**	R2 = 0.16; F = 4.29; p = **0.004**
			Overall treatment effect per timepoint		
**Bray-Curtis**	*Pre-treatment*	*2 weeks*	*5 weeks*	*9 weeks*	*14 weeks*
ASV	R2 = 0.13; F = 0.72; p = 0.842	R2 = 0.17; F = 0.95; p = 0.640	R2 = 0.19; F = 1.17; p = 0.228	R2 = 0.15; F = 0.82; p = 0.857	R2 = 0.15; F = 0.85; p = 0.819
Order	R2 = 0.12; F = 0.69; p = 0.810	R2 = 0.13; F = 0.72; p = 0.997	R2 = 0.18; F = 1.07; p = 0.598	R2 = 0.09; F = 0.47; p = 0.997	R2 = 0.09; F = 0.46; p = 0.997
			Overall time effect per treatment		
	Control	*$nTiO_2$*	*Carbendazim*	*Combined*	*$nTiO_2$-coated CBZ*
ASV	R2 = 0.09; F = 2.2; p = **0.002**	R2 = 0.1; F = 2.5; p = **0.002**	R2 = 0.09; F = 1.98; p = **0.002**	R2 = 0.1; F = 2.51; p = **0.002**	R2 = 0.09; F = 2.27; p = **0.002**
Order	R2 = 0.13; F = 3.3; p = 0.080	R2 = 0.11; F = 2.89; p = 0.080	R2 = 0.08; F = 1.79; p = 0.140	R2 = 0.14; F = 3.59; p = **0.030**	R2 = 0.08; F = 1.98; p = 0.194

Note: Statistically significant effects are indicated by bold p-values. Combined = Carbendazim & $nTiO_2$; CBZ = carbendazim. No significant differences were observed in betadispersion.

List of figures

S1. Monthly averages (mean ± standard error) of physicochemical water quality parameters measured over the course of the experiment. Note that chlorophyll A and turbidity are measured in arbitrary units. This data has previously been reported in ref. 4. 15

S2. Concentrations (mean ± standard error) of applied treatments across the timeframe of the experiment measured in samples collected from the water column for (A) nTiO2, (B) Carbendazim, (C) the combined treatment (nTiO2 & Carbendazim) and (D) the sum of freely dissolved and nTiO2-coated carbendazim, as well as the cumulative release of carbendazim from the nTiO2-coated formulation in demineralized water assessed in vitro. This data has previously been reported in ref. 4. 16

S3. Rarefaction curves for each of the markers. The vertical line indicates the cut-off point where most samples reached a plateau. This value is indicated between brackets per marker. (A) 16S (plateau at 10,000 reads); (B) 18S (plateau at 6,000 reads); (C) ITS2 (plateau at 6,000 reads). Note that axes are on different scales to fit all samples for each marker. 17

S4. Boxplots showing relative read abundance per sample, visualized per phylum (or class within phylum for Proteobacteria) for the bacteria data. Note that y-axes are on different scales to fit the data. 18

S5. Boxplots visualizing bacteria alpha-diversity between treatments over time. (A) ASV richness; (B) Family richness; (C) Read-abundance-based Shannon Weiner index scores based on unassigned ASVs; (D) Read-abundance-based Shannon Weiner index scores based on annotated family level data. Note that x-axes are on different scales to fit the data. 19

S6. Boxplots visualizing ASV richness per phylum (or class within phylum for Proteobacteria) for the bacteria data. Note that y-axes are on different scales to fit the data. 20

S7. Principle coordinate analyses (PCoA) plots visualizing bacteria beta-diversity over time. (A) ASV-based Sørensen (dis)similarity; (B) Family-based Sørensen (dis)similarity; (C) ASV-based Bray-Curtis (dis)similarity; (D) Family-based Bray-Curtis (dis)similarity. Centroids represent mean PCoA scores per treatment and polygons are drawn around PCoA scores of individual replicates (i.e., ditches). 21

S8. Boxplots visualizing relative read abundance, visualized per phylum (or order within phylum for Ochrophyta) for the phytoplankton data. Note that the y-axes are on different scales to fit the data. 22

S9. Boxplots visualizing phytoplankton alpha-diversity between treatments over time. (A) ASV richness; (B) Order richness; (C) Read-abundance-based Shannon Weiner index scores based on unassigned ASVs; (D) Read-abundance-based Shannon Weiner index scores based on annotated order level data. Note that x-axes are on different scales to fit the data. 23

S10. Boxplots visualizing ASV richness per phylum (or order within phylum for Ochrophyta) for the phytoplankton data. Note that y-axes are on different scales to fit the data. 24

S11. Principle coordinate analyses (PCoA) plots visualizing phytoplankton beta-diversity over time. (A) ASV-based Sørensen (dis)similarity; (B) Order-based Sørensen (dis)similarity; (C) ASV-based Bray-Curtis (dis)similarity; (D) Order-based Bray-Curtis (dis)similarity. Centroids represent mean PCoA scores per treatment and polygons are drawn around PCoA scores of individual replicates (i.e., ditches). 25

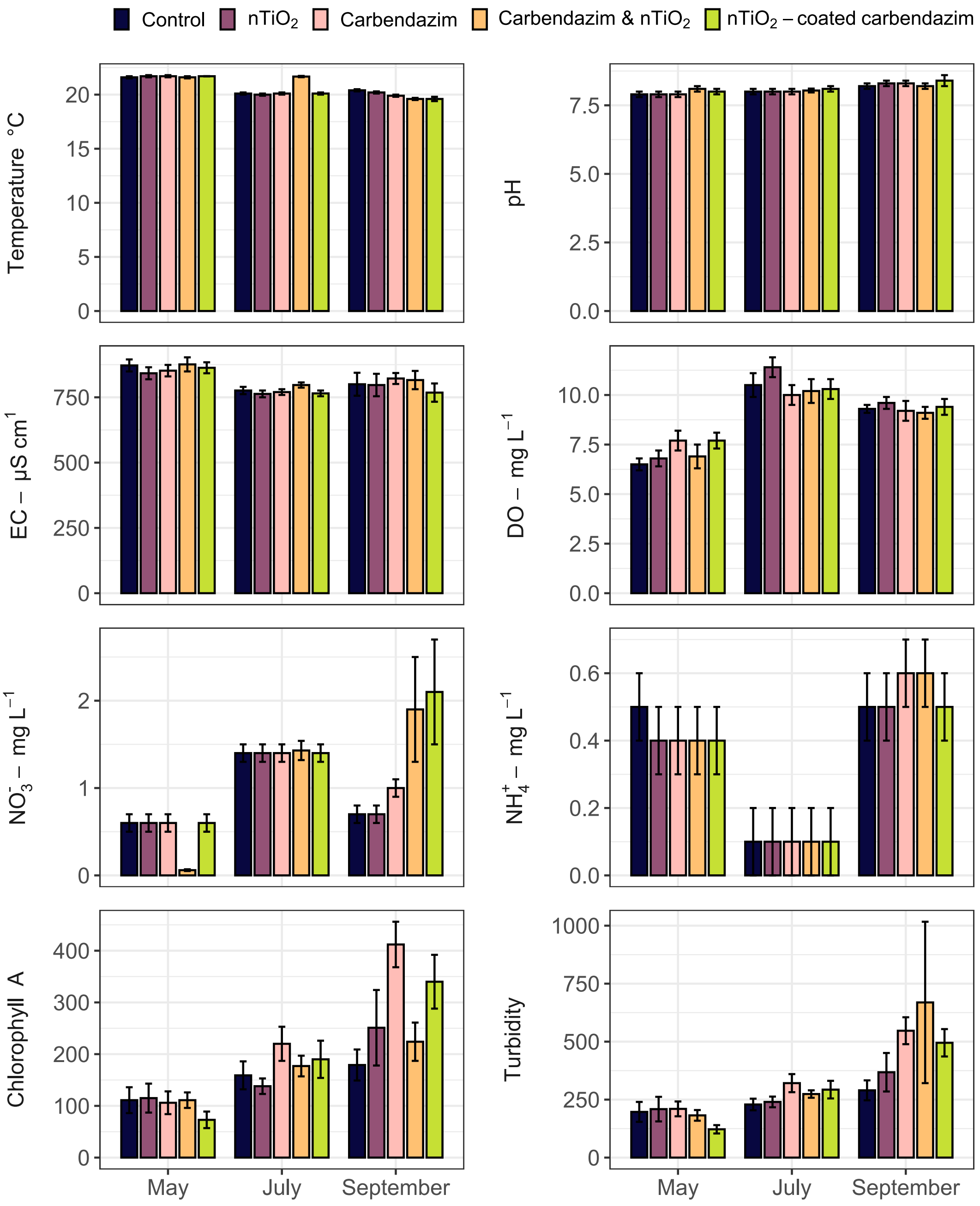

Supporting figure S1. Monthly averages (mean ± standard error) of physicochemical water quality parameters measured over the course of the experiment. Note that chlorophyll A and turbidity are measured in arbitrary units. This data has previously been reported in ref. 18.

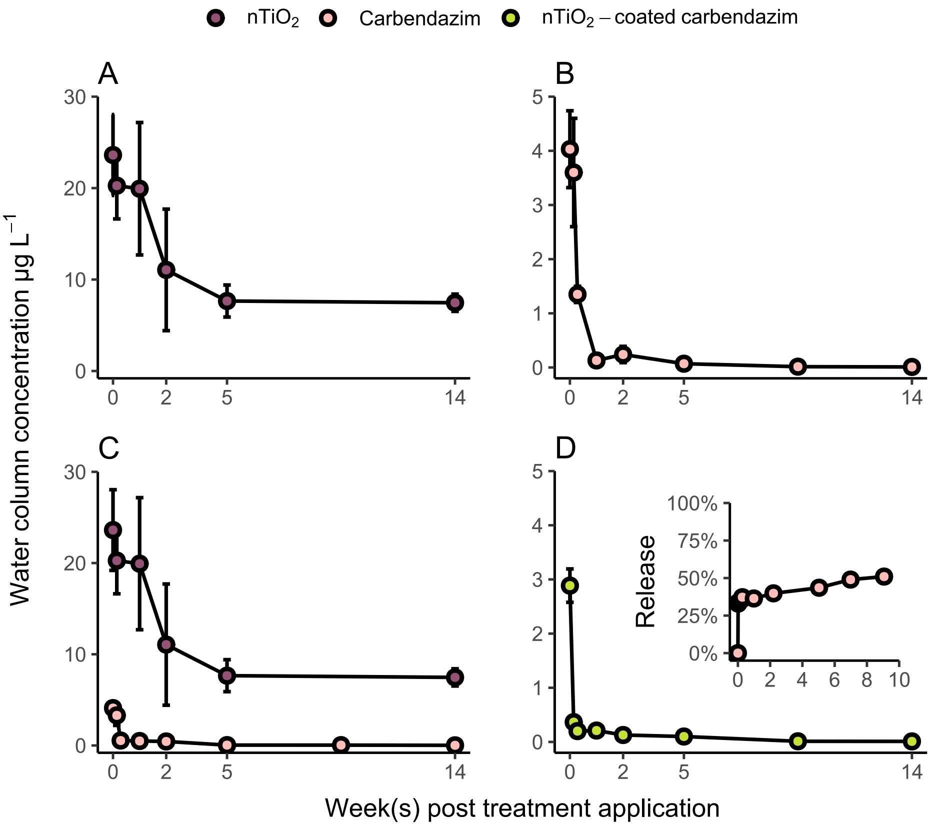

Supporting figure S2. Concentrations (mean ± standard error) of applied treatments across the timeframe of the experiment measured in samples collected from the water column for (A) $nTiO_2$, (B) Carbendazim, (C) the combined treatment ($nTiO_2$ & Carbendazim) and (D) the sum of freely dissolved and $nTiO_2$-coated carbendazim, as well as the cumulative release of carbendazim from the $nTiO_2$-coated formulation in demineralized water assessed *in vitro*. This data has previously been reported in ref. 18.

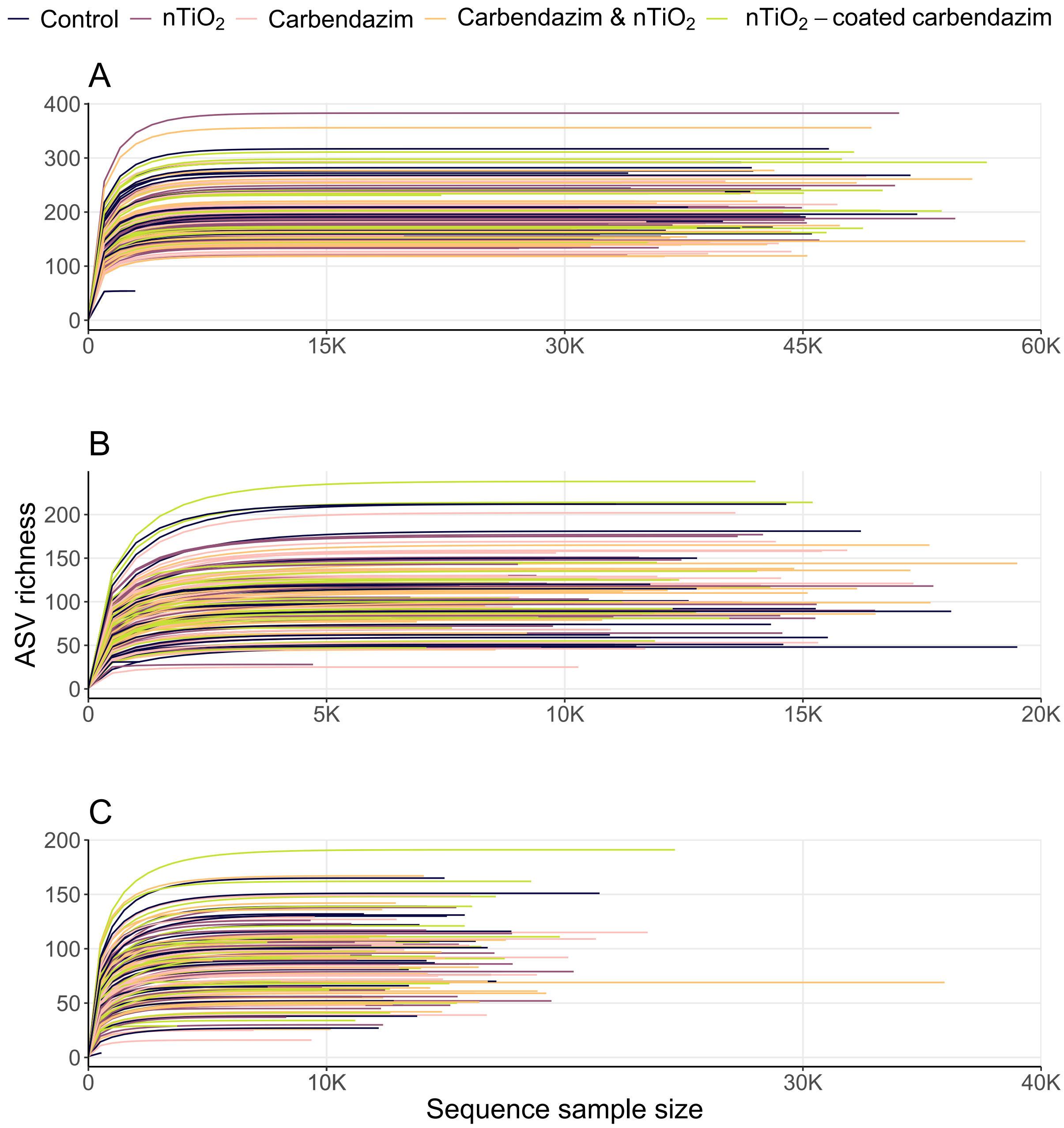

Supporting figure S3. Rarefaction curves for each of the markers. The vertical line indicates the cut-off point where most samples reached a plateau. This value is indicated between brackets per marker. (A) 16S (plateau at 10,000 reads); (B) 18S (plateau at 6,000 reads); (C) ITS2 (plateau at 6,000 reads). Note that axes are on different scales to fit all samples for each marker.

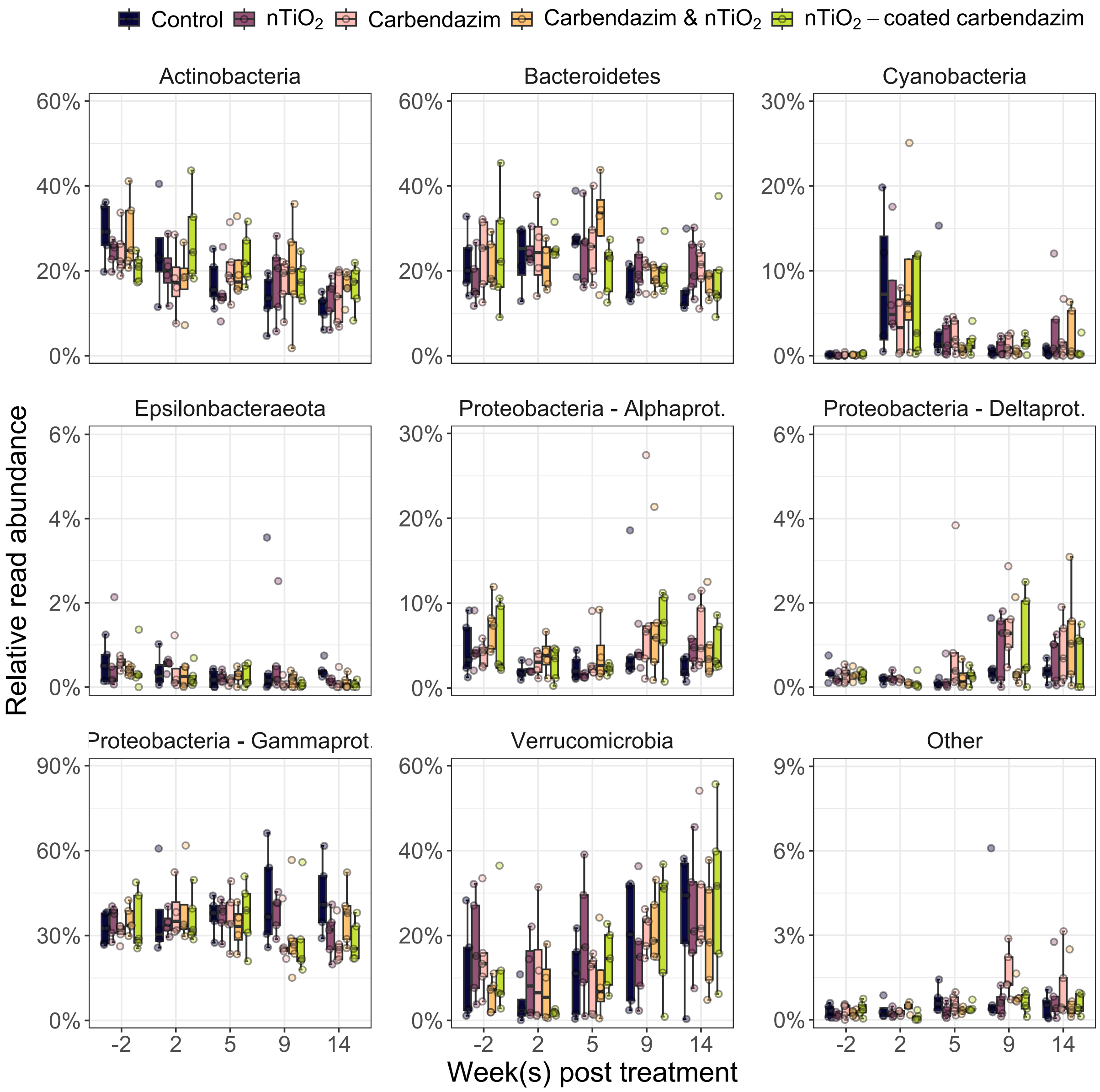

Supporting figure S4. Boxplots showing relative read abundance per sample, visualized per phylum (or class within phylum for *Proteobacteria*) for the bacteria data. Note that y-axes are on different scales to fit the data.

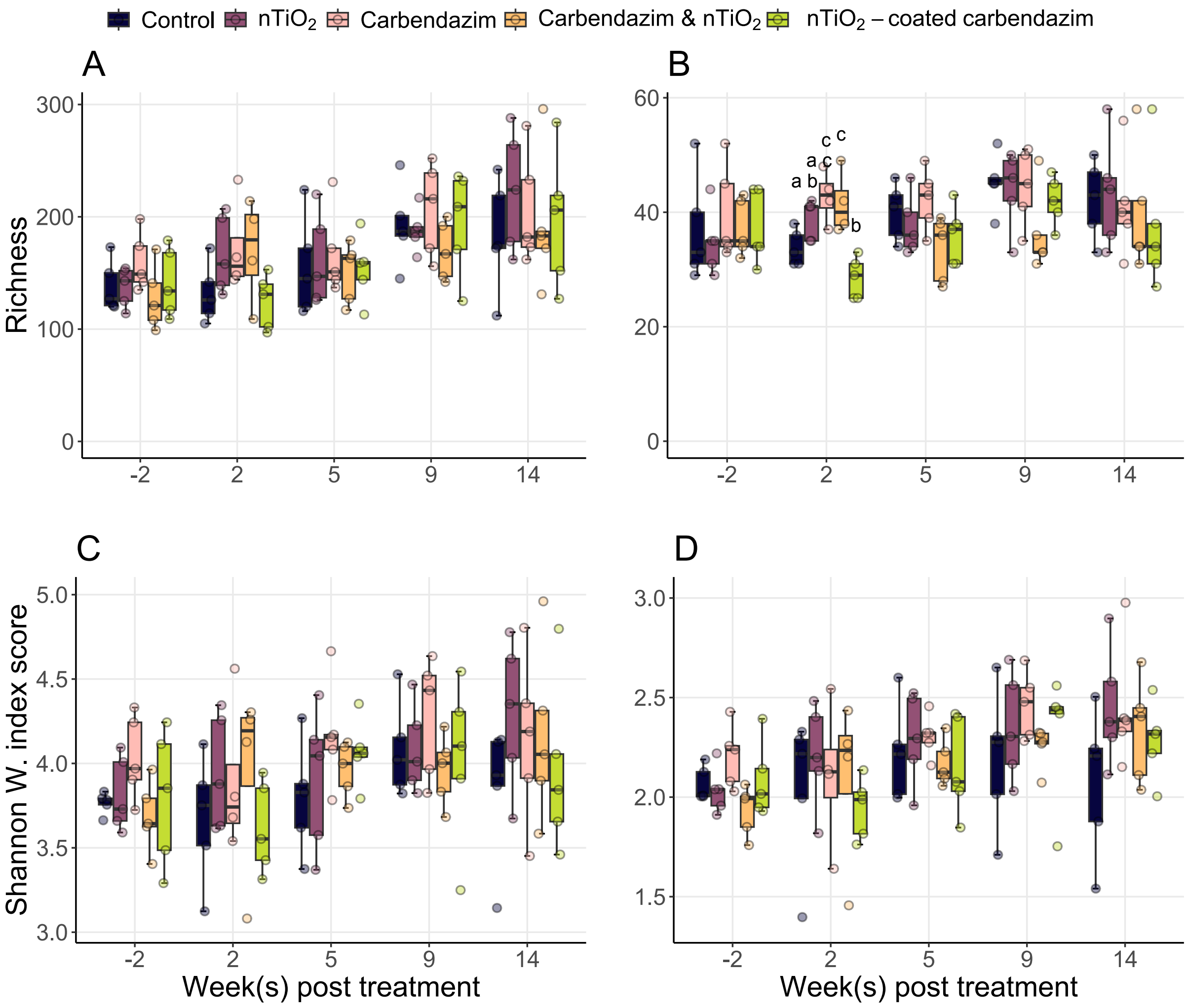

Supporting figure S5. Boxplots visualizing bacteria alpha-diversity between treatments over time. (A) ASV richness; (B) Family richness. Significant differences between treatments within timepoint 2 are indicated by letters, with boxes sharing a letter being not significantly different from one another; (C) Read-abundance-based Shannon Weiner index scores based on unassigned ASVs; (D) Read-abundance-based Shannon Weiner index scores based on annotated family level data. Note that x-axes are on different scales to fit the data.

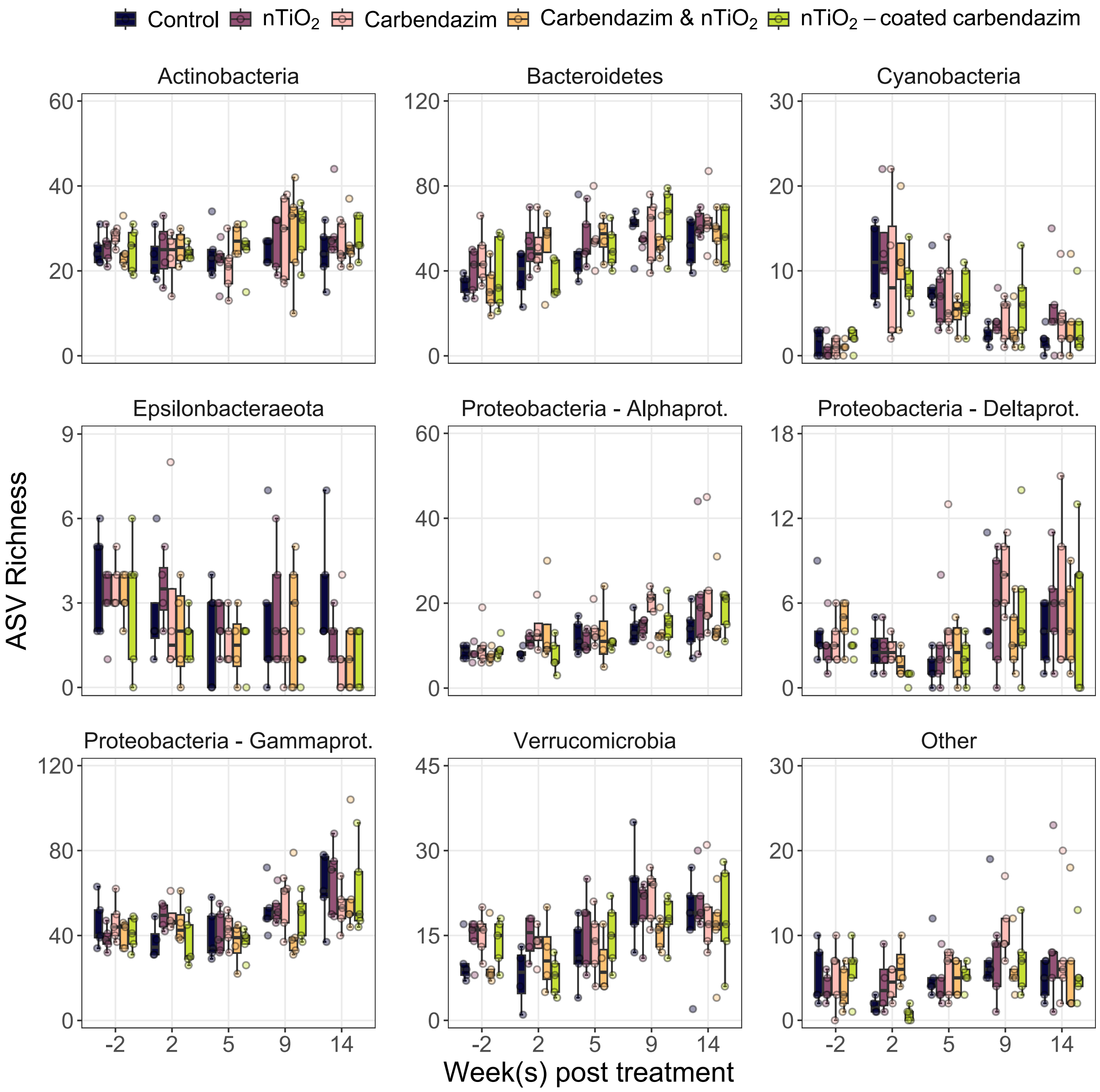

Supporting figure S6. Boxplots visualizing ASV richness per phylum (or class within phylum for *Proteobacteria*) for the bacteria data. Note that y-axes are on different scales to fit the data.

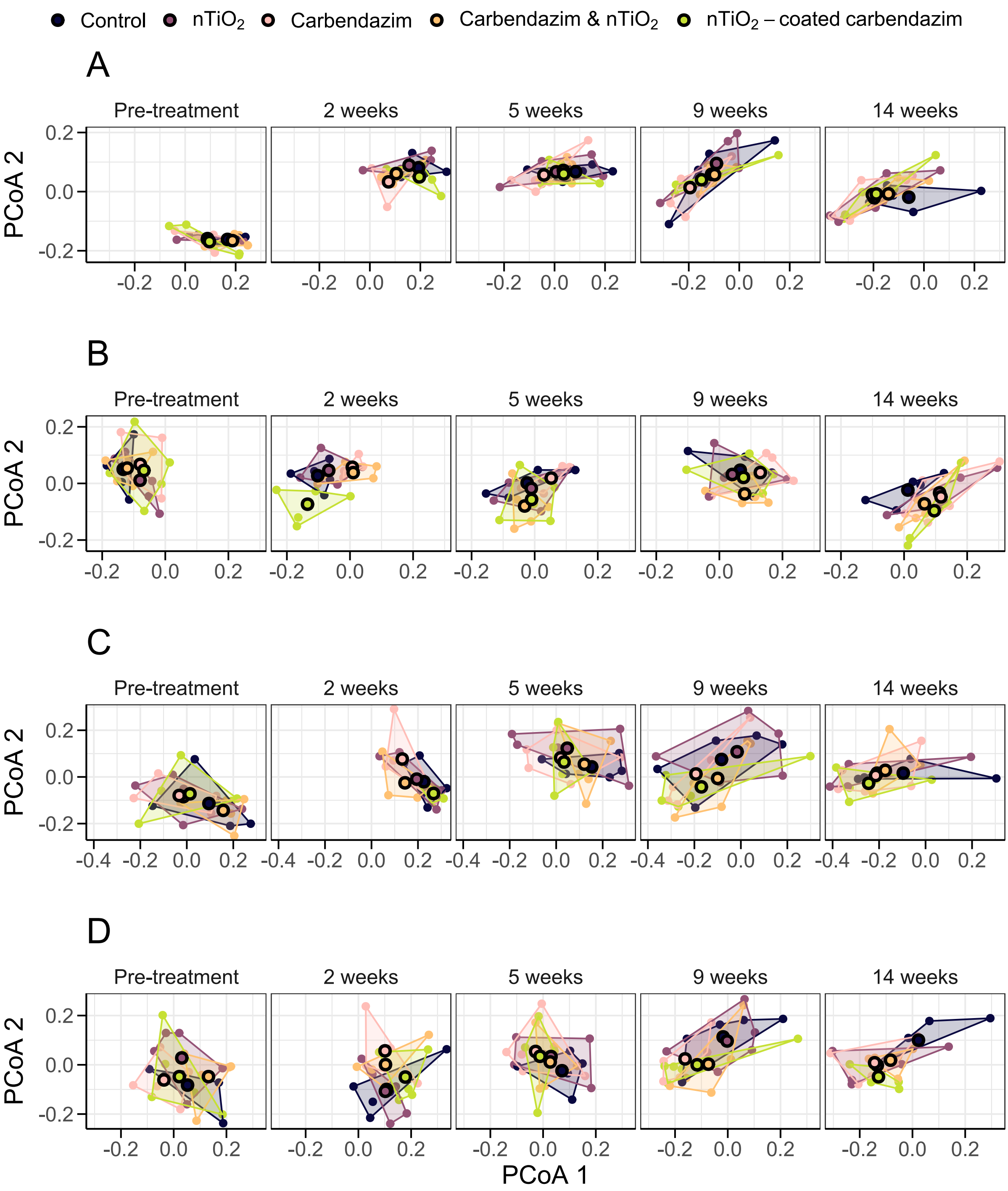

Supporting figure S7. Principle coordinate analyses (PCoA) plots visualizing bacteria beta-diversity over time. (A) ASV-based Sørensen (dis)similarity; (B) Family-based Sørensen (dis)similarity; (C) ASV-based Bray-Curtis (dis)similarity; (D) Family-based Bray-Curtis (dis)similarity. Centroids represent mean PCoA scores per treatment and polygons are drawn around PCoA scores of individual replicates (i.e., ditches). Note that axes scales differ between plots.

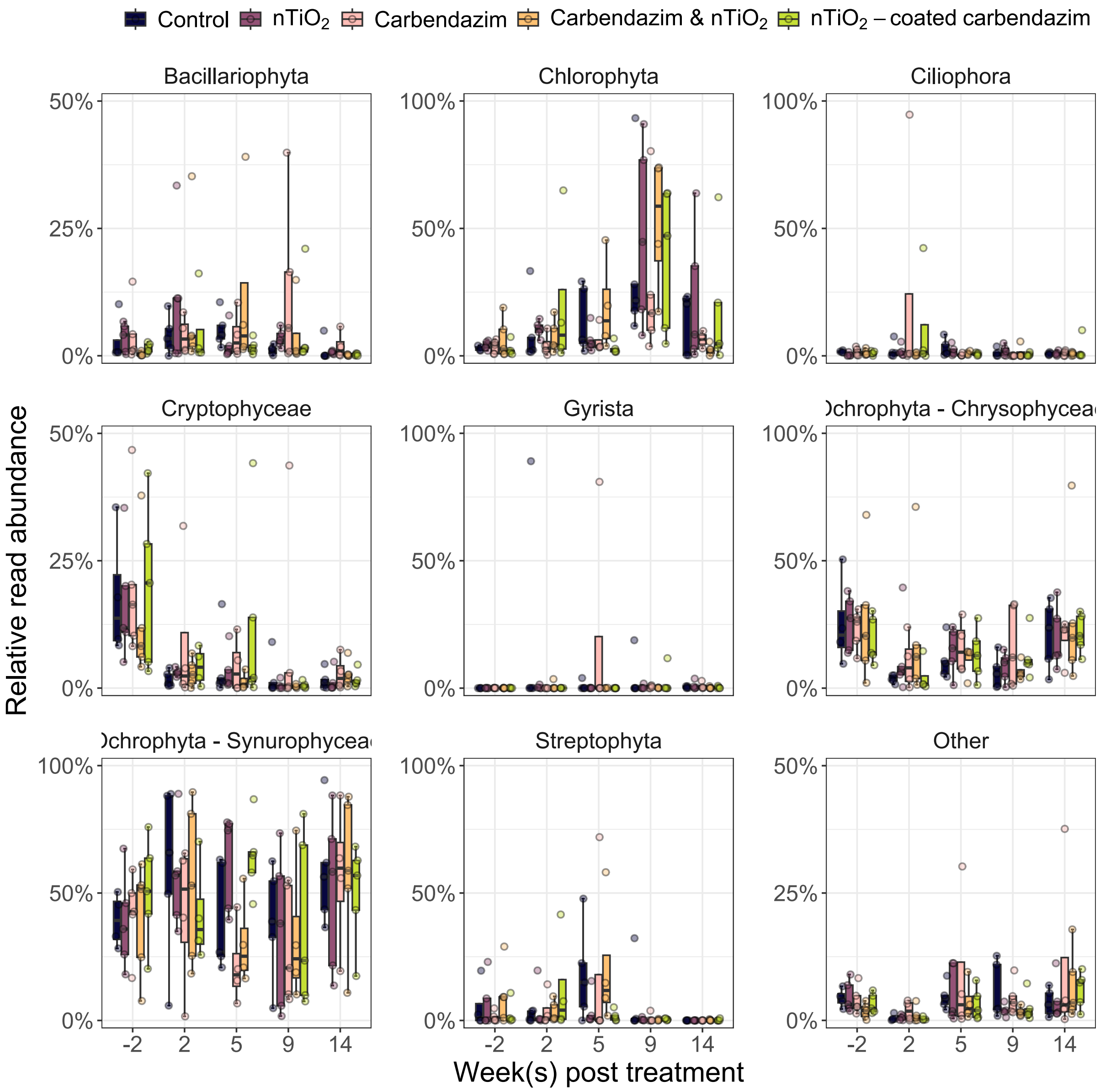

Supporting figure S8. Boxplots visualizing relative read abundance, visualized per phylum (or order within phylum for *Ochrophyta*) for the phytoplankton data. Note that the y-axes are on different scales to fit the data.

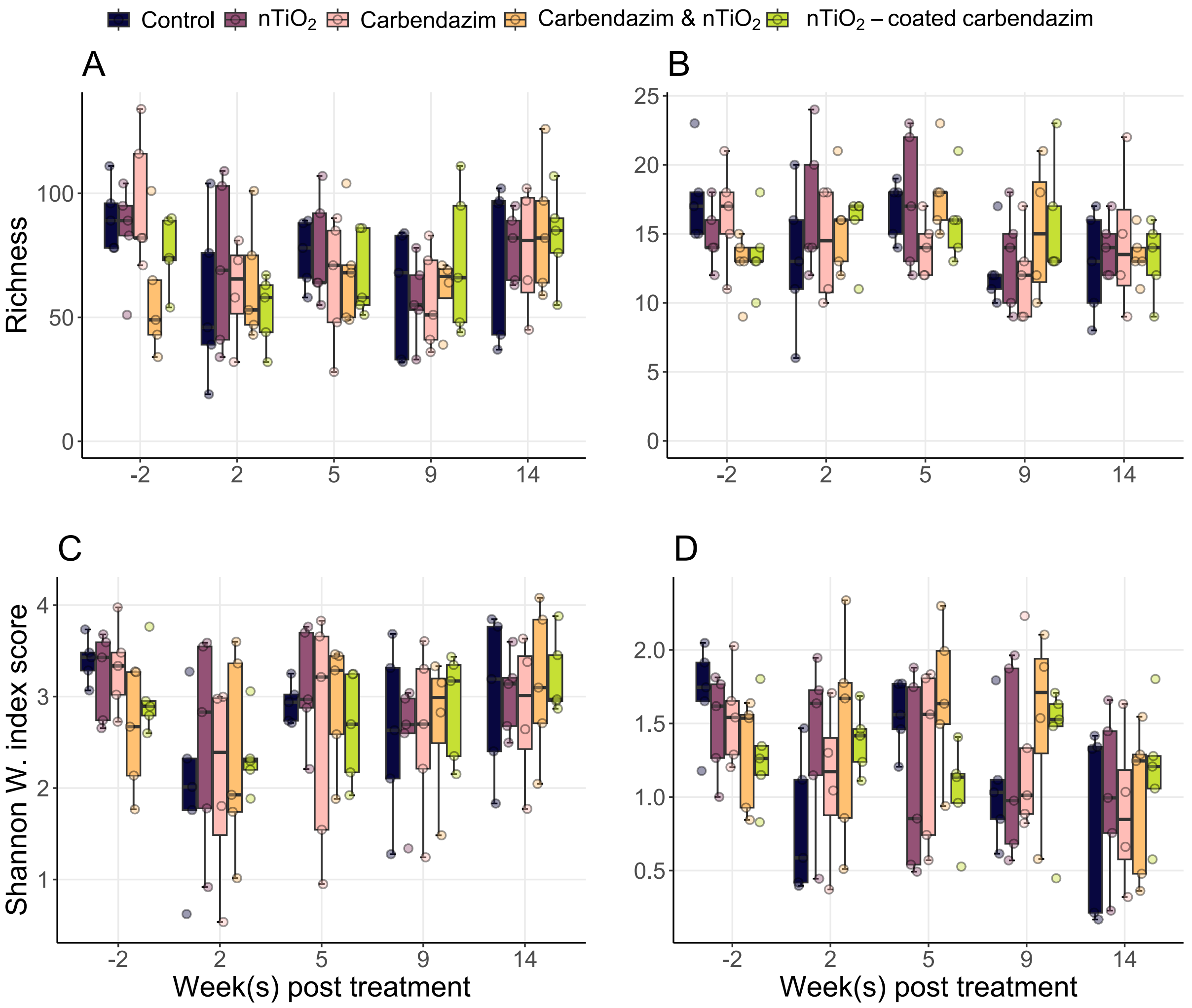

Supporting figure S9. Boxplots visualizing phytoplankton alpha-diversity between treatments over time. (A) ASV richness; (B) Order richness; (C) Read-abundance-based Shannon Weiner index scores based on unassigned ASVs; (D) Read-abundance-based Shannon Weiner index scores based on annotated order level data. Note that x-axes are on different scales to fit the data.

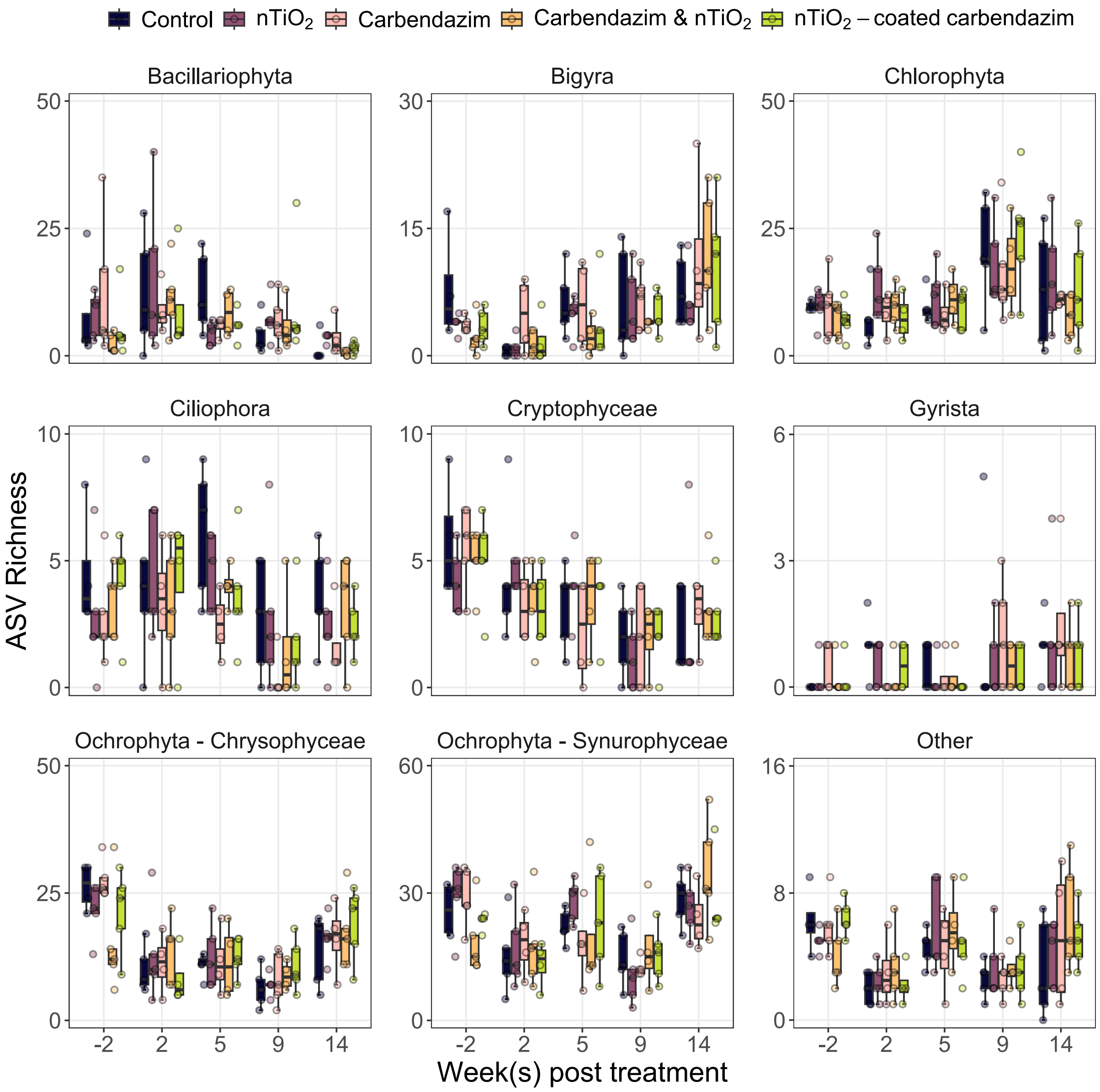

Supporting figure S10. Boxplots visualizing ASV richness per phylum (or order within phylum for *Ochrophyta*) for the phytoplankton data. Note that y-axes are on different scales to fit the data.

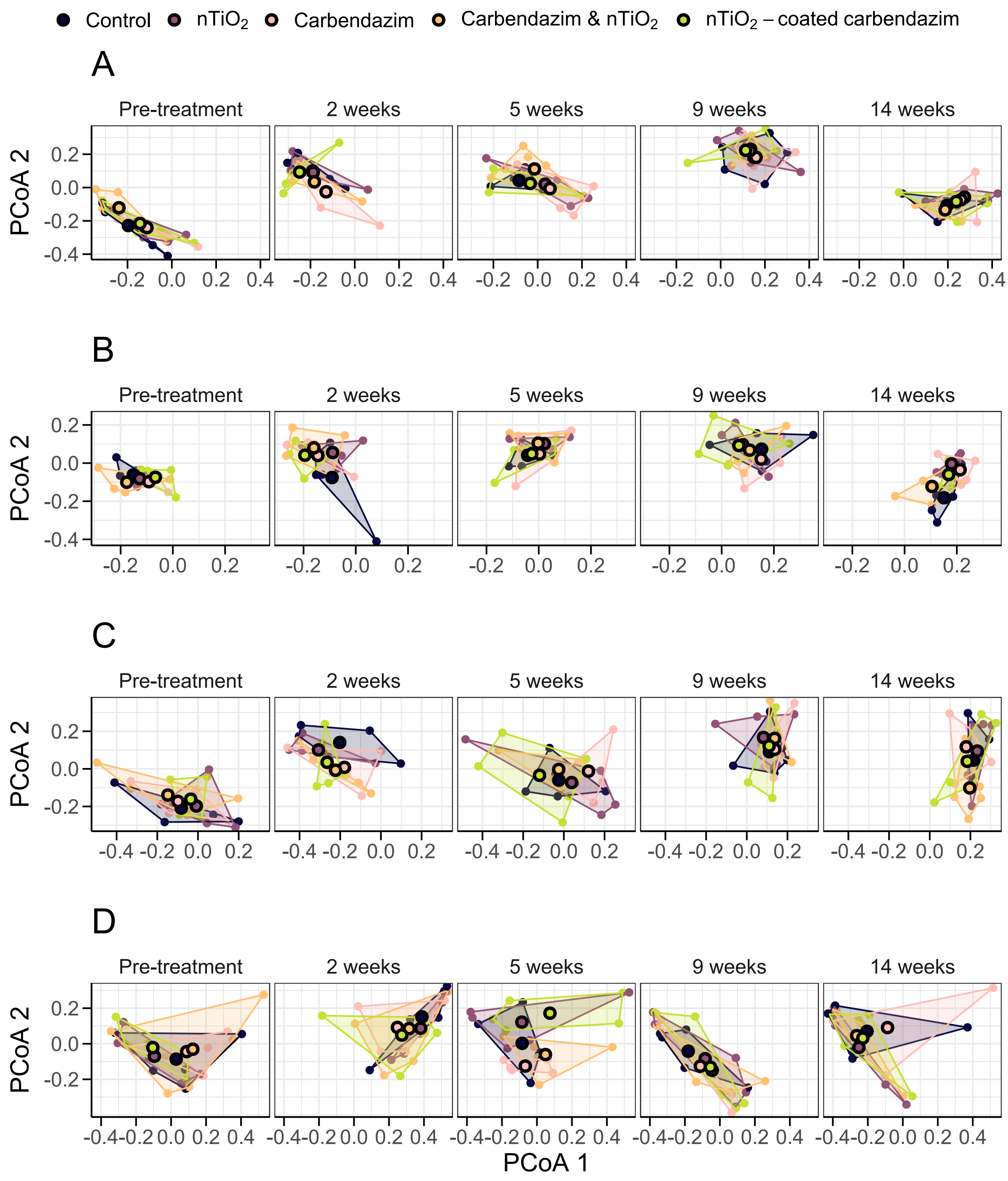

Supporting figure S11. Principle coordinate analyses (PCoA) plots visualizing phytoplankton beta-diversity over time. (A) ASV-based Sørensen (dis)similarity; (B) Order-based Sørensen (dis)similarity; (C) ASV-based Bray-Curtis (dis)similarity; (D) Order-based Bray-Curtis (dis)similarity. Centroids represent mean PCoA scores per treatment and polygons are drawn around PCoA scores of individual replicates (i.e., ditches). Note that axes scales differ between plots.

References

1. Kumar, G., Farrell, E., Reaume, A. M., Eble, J. A. & Gaither, M. R. One size does not fit all: Tuning eDNA protocols for high- and low-turbidity water sampling. *Environ. DNA* **4**, 167–180 (2022).

2. Byappanahalli, M. N. *et al.* Influence of Filter Pore Size on Composition and Relative Abundance of Bacterial Communities and Select Host-Specific MST Markers in Coastal Waters of Southern Lake Michigan. *Front. Microbiol.* **12**, 1–11 (2021).

3. Invasive crayfish: drivers or passengers of degradation in freshwater ecosystems? *Environ. DNA*.

4. Beentjes, K. K. *et al.* Environmental DNA metabarcoding reveals comparable responses to agricultural stressors on different trophic levels of a freshwater community. *Mol. Ecol.* **31**, 1430–1443 (2021).

5. Klindworth, A. *et al.* Evaluation of general 16S ribosomal RNA gene PCR primers for classical and next-generation sequencing-based diversity studies. *Nucleic Acids Res.* **41**, 1–11 (2013).

6. Zimmermann, J., Jahn, R. & Gemeinholzer, B. Barcoding diatoms: Evaluation of the V4 subregion on the 18S rRNA gene, including new primers and protocols. *Org. Divers. Evol.* **11**, 173–192 (2011).

7. White, T. J., Bruns, T., Lee, S. & Taylor, J. Amplification and direct sequencing of fungal ribosomal RNA Genes for phylogenetics. in *PCR - Protocols and Applications - A Laboratory Manual* 315–322 (Academic Press, 1990).

8. Bolyen, E. *et al.* Reproducible, interactive, scalable and extensible microbiome data science using QIIME 2. *Nat. Biotechnol.* **37**, 852–857 (2019).

9. Martin, M. Cutadapt removes adapter sequences from high-throughput sequencing reads. *EMBnet journal2* **17**, pp-10 (2011).

10. Callahan, B. J. *et al.* DADA2: High-resolution sample inference from Illumina amplicon data. *Nat. Methods* **13**, 581–583 (2016).

11. Bokulich, N. A. *et al.* Optimizing taxonomic classification of marker-gene amplicon sequences with QIIME 2's q2-feature-classifier plugin. *Microbiome* **6**, 1–17 (2018).

12. Pedregosa, F. *et al.* Scikit-learn: Machine Learning in Python. *J. ofMachine Learn. Res.* **12**, 2825–2830 (2011).

13. Quast, C. *et al.* The SILVA ribosomal RNA gene database project: Improved data processing and web-based tools. *Nucleic Acids Res.* **41**, 590–596 (2013).

14. Abarenkov, K. *et al.* UNITE QIIME release for eukaryotes 2. *UNITE Community* (2021) doi:10.15156/BIO/1264861.

15. Drake, L. E. *et al.* An assessment of minimum sequence copy thresholds for identifying and reducing the prevalence of artefacts in dietary metabarcoding data. *Methods Ecol. Evol.* **13**, 694–710 (2022).

16. Dueholm, M. S. *et al.* Generation of comprehensive ecosystem-specific reference databases with species-level resolution by high-throughput full-length 16s rrna gene sequencing and automated taxonomy assignment (Autotax). *MBio* **11**, 1–14 (2020).

17. Heino, J. & Soininen, J. Are higher taxa adequate surrogates for species-level assemblage

patterns and species richness in stream organisms? *Biol. Conserv.* **137**, 78–89 (2007).

18. Schloss, P. D. Removal of rare amplicon sequence variants from 16S rRNA gene sequence surveys biases the interpretation of community structure data. *bioRxiv* 2020.12.11.422279 (2020).

19. Alberdi, A., Aizpurua, O., Gilbert, M. T. P. & Bohmann, K. Scrutinizing key steps for reliable metabarcoding of environmental samples. *Methods Ecol. Evol.* **9**, 134–147 (2018).

20. Zinger, L. *et al.* DNA metabarcoding—Need for robust experimental designs to draw sound ecological conclusions. *Mol. Ecol.* **28**, 1857–1862 (2019).

18. Nederstigt, T. A. P., Peijnenburg, W. J. G. M., Schrama, M., van Ommen, J. R. & Vijver, M. G. Impacts of a novel controlled-release TiO2-coated (nano-) formulation of carbendazim and its constituents on freshwater macroinvertebrate communities. *Sci. Total Environ.* **838**, 156554 (2022).
